# Synthesis and Molecular Characterization of Pyrene-Containing Copolymers as Potential MALDI-TOF MS Matrices

**DOI:** 10.1021/acs.macromol.5c00492

**Published:** 2025-07-09

**Authors:** Marileta Tsakanika, Eleni Aleiferi, Dimitrios Damalas, Anastasia Stergiou, Nikolaos S. Thomaidis, Georgios Sakellariou

**Affiliations:** † Laboratory of Industrial Chemistry, Department of Chemistry, 68993 National and Kapodistrian University of Athens, Panepistimiopolis, Zografou, 15771 Athens, Greece; ‡ Laboratory of Analytical Chemistry, Department of Chemistry, 68993 National and Kapodistrian University of Athens, Panepistimiopolis, Zografou, 15771 Athens, Greece

## Abstract

Due to its exceptional sensitivity, accuracy, and speed, matrix-assisted laser desorption/ionization time-of-flight (MALDI-TOF) mass spectrometry has emerged as a vital analytical tool, especially for the determination of low-molecular-weight compounds (e.g., lipids, metabolites). Continuous advancements in MALDI-TOF technology have expanded its applications. The employment of polymeric materials as matrices has proven to be effective in overcoming significant challenges (self-ionization and adduct formation), particularly those related to interfering background signals in the low-molecular-weight region, making the development of effective matrices a critical area of research. We investigated the synthesis of well-defined polymers that meet the requirements of a suitable matrix for MALDI-TOF MS. In this study, pyrene was chosen as a chromophore to enhance the optical properties of the polymers, taking advantage of its aromatic structure and prominent absorption capabilities. We present the detailed synthesis of novel linear polymers through reversible addition–fragmentation chain transfer polymerization, which afforded macromolecules in a controlled manner and with narrow dispersity (*Đ*). Reactivity ratios were calculated to provide insight into the copolymerization behavior, allowing precise control over the polymer composition. Finally, these pyrene-incorporating polymers were tested and evaluated for their applicability as MALDI-TOF matrices, particularly in the analysis of low-molecular-weight compounds. Their performance was assessed based on analyte signal intensities and in comparison with other commercially available polymeric matrices (P3DDT), highlighting their potential as robust tools for mass spectrometric analysis.

## Graphical abstract

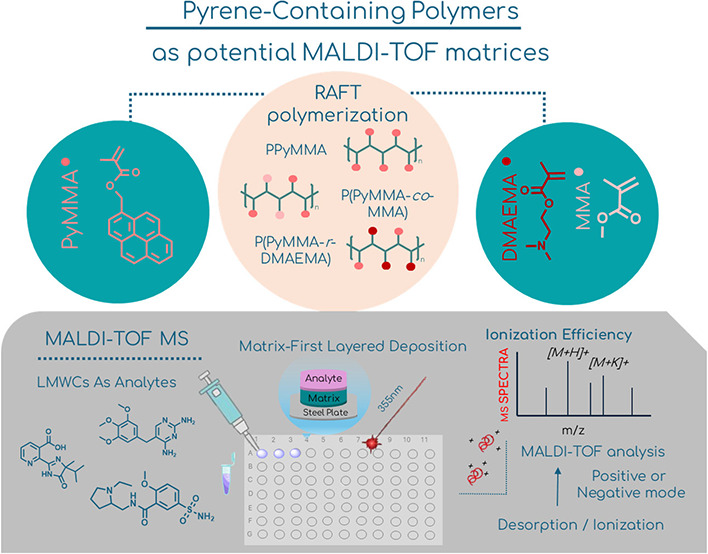

## Introduction

The advent of matrix-assisted laser desorption/ionization time-of-flight mass spectrometry (MALDI-TOF MS) has led to transformative advancements in the mass spectrometric analysis of biomolecules and synthetic polymers. Originally developed through the pioneering works of Tanaka,^1^ who first demonstrated MALDI for large molecule analysis, and further refined by Karas^2^ and Hillenkamp,^3,4^ this technique enabled the study of large, labile molecules with minimal fragmentation. MALDI-TOF MS allowed for the analysis of proteins, peptides, and synthetic polymers with remarkable ease and sensitivity, providing a breakthrough in fields that require the characterization of complex macromolecules.^5–8^

Today, MALDI-TOF MS extends beyond macromolecules to meet an increasing demand for the analysis of low-molecular-weight compounds (LMWCs),^9–11^ such as pharmaceuticals, metabolites, and environmental toxins. This shift is driven by the need for rapid, precise analysis in areas, including pharmacology, environmental monitoring, and quality control, where even minor variations in composition can be significant. MALDI-TOF MS, with its capability for sensitive, high-throughput analysis, has become an indispensable tool for understanding, developing, and managing these critical compounds.^12^

The choice of the matrix in a typical MALDI experiment is crucial since it directly influences both the desorption and ionization processes, thus affecting the quality of the resulting mass spectra.^13^ Selection of the matrix is dependent on the chemical nature^14^ of the analyte, as well as on a number of key performance criteria. First, the matrix must strongly absorb at the working wavelength of the laser, usually in the UV range, for efficient energy transfer. It should also exhibit high ionization efficiency to ensure strong analyte ion signals^15^ and therefore sensitive detection. Furthermore, the matrix must be chemically inert toward the analyte, preventing unwanted interactions that could affect the integrity of the analyte signal. Vacuum stability is also a requirement, as MALDI systems typically operate under low-pressure conditions.^16^ Intrinsic noise from the matrix must be minimized to reduce interference and ensure a clear and distinct signal from the analyte. Effective incorporation of the analyte within the matrix and the quality of the thin film generated during the sample preparation are highly significant. Currently, small organic molecules (SOMs) are predominantly used as MALDI-TOF MS matrices. Bearing π-conjugated systems to achieve laser absorption and functional groups, mainly carboxyl groups, to promote ionization efficiency, compounds such as dihydroxybenzoic acid (DHB) and α-cyano-4-hydroxycinnamic acid (α-CHCA) offer strong performance for many analytical applications. However, when analyzing LMWCs, these matrices present significant drawbacks. Self-desorption and ionization often generate numerous matrix-related peaks in the low-mass range, obstructing peaks of the analytical target and complicating accurate analysis.^10^

So far, the search for new compounds that could effectively serve as MALDI-TOF matrices has been a trial-and-error process.^17,18^ In order to address the limitations of SOMs in LMWC analysis, several novel approaches have been developed. For example, Abdelhamid^19^ converted SOMs into ionic liquids (ILs) by forming acid–base conjugate pairs, resulting in matrices that leverage the inherent vacuum stability of ILs while exhibiting reduced matrix peak interferences in the low-mass range. Another strategy involves attaching SOMs to much larger, complementary structures to increase molecular weight, shifting matrix peaks away from the low-mass range and thereby increasing analyte peak resolution.^20^

Recently, Horatz et al.^21^ proposed a new approach to modify conventional SOMs by introducing vinyl groups into compounds such as DHB and harmine (Har). This modification enabled free-radical polymerization, incorporating SOMs as pendant groups along the polymer chain. The resulting polymeric matrices, namely, (P­(VDHB)) and (P­(VHar)), retained most of the desirable properties of the original SOMs but reduced matrix-related signals substantially.

Utilizing the unique properties of polymers in the MALDI-TOF matrix design unlocks vast potential for innovation. With their diverse structures, polymers can allow a wider range of functionalizations, increasing their specificity or customization for the matrix development. Higher molecular weight matrices could offer greater vacuum stability but could also help reduce interference by remaining “MALDI silent”, i.e., do not give matrix-related signals in the LMW area. Well-established targeted modification reactions allow for fine-tuning of substituents which can shift both absorption maxima and ionization potentials.^18^ In addition, polymers offer chemical inertness, making them stable and resistant to undesired reactions during the analysis. By the appropriate adjustment of their chemical structure, it is possible to precisely control polymer morphology, further optimizing the matrix performance. Given these promising attributes, there are still relatively few studies in which these features are fully explored or implemented in the MALDI-TOF matrix design.

Recently, Horatz et al.^22,23^ published two follow-up studies in which a series of amorphous copolymers, including poly­{[N,N’-bis­(2-octyldodecyl)-naphthalene-1,4,5,8-bis­(dicarboximide)-2,6-diyl]-*alt*-5,5′(2,2’-bithiophene)} (PNDI­(T2)), poly­(3-dodecylthiophene-2,5-diyl) (P3DDT), poly­{[2,3-bis­(3-octyloxyphenyl)­quinoxaline-5,8-diyl]-*alt*-(thiophene-2,5-diyl)} (PTQ1), poly­{[N,N’-bis­(2-octyldodecyl)-isoindigo-5,5′-diyl]-*alt*-5,5′(2,2’-bithiophene)} (PII­(T2)), poly­(9,9-di-n-octylfluorenyl-2,7-diyl) (P9OFl), P­(TNDIT–FI­(C10C8)), and P­(TNDIT–FI­(C10C8)), was synthesized as MALDI matrices for the detection of LMWCs in both positive and negative ionization modes. According to their investigation on the relationship between matrix crystallinity and performance, amorphous matrices performed noticeably better than semicrystalline ones in terms of the LMWC signal intensity detection. Motivated by these results, Chen et al.^24^ used totally amorphous poly­(9-vinylcarbazole) (PVK) as a matrix in a negative ionization mode to detect a variety of small compounds.

In this work, a series of copolymers was designed and synthesized by incorporating the pyrene chromophore as a side group onto the polymer main chain. Pyrene exhibits strong absorption in the UV region and has been used extensively in various optoelectronic applications, suggesting the presence of efficient electron transfer reactions^25^ that may be advantageous for the ionization of LMWCs. Initially, homopolymers of poly­(1-pyrenyl methyl methacrylate) (PPyMMA) were synthesized by RAFT polymerization, followed by the synthesis of random copolymers with methyl methacrylate (MMA) and 2-dimethylaminoethyl methacrylate (DMAEMA), chosen as neutral and basic monomers, respectively. The effect of the pyrene chromophore, in conjunction with the functional groups, was also investigated to determine whether their presence altered optical and thermal properties of the materials as well as their influence on morphology and potential MALDI-TOF performance.

## Experimental Section

### Synthesis of 1-Pyrenylmethyl Methacrylate (PyMMA)

The synthesis of 1-pyrenylmethyl methacrylate was carried out via a nucleophilic acyl substitution reaction between 1-pyrenemethanol and methacryloyl chloride (Figure 1). A solution of 1-pyrenemethanol (1 g, 4 mmol) in a mixture of distilled triethylamine (1.9 mL, 13 mmol) and tetrahydrofuran (10 mL) was prepared. The solution was degassed, stirred until homogeneous, and then transferred to the Schlenk line and filled with inert gas. For the final step, the addition of methacryloyl chloride (1 mL, 10 mmol), a dropping funnel prefilled with inert gas, was attached to the flask. Given the highly exothermic nature of the reaction between methacryloyl chloride and 1-pyrenemethanol, it was essential to conduct the reaction at a low temperature; therefore, the setup was immersed in an ice–water bath. When the target temperature (0 °C) was reached, methacryloyl chloride was added dropwise using the cannula technique to avoid atmospheric exposure, as it is highly reactive in air. The reaction mixture was stirred for 10 min and then removed from the ice bath. Upon the addition of methacryloyl chloride, the solution color changed from yellowish to dark yellow. The mixture was subsequently stirred overnight. Upon completion of the reaction, the mixture was filtered through a porous filter to remove the triethylamine hydrochloride salts formed, and the filtrate was concentrated to dryness. The resulting product was redissolved in tetrahydrofuran and precipitated in methanol. Finally, the product was freeze-dried in benzene, yielding a yellowish powder (yield = 50%)

**Figure 1:**
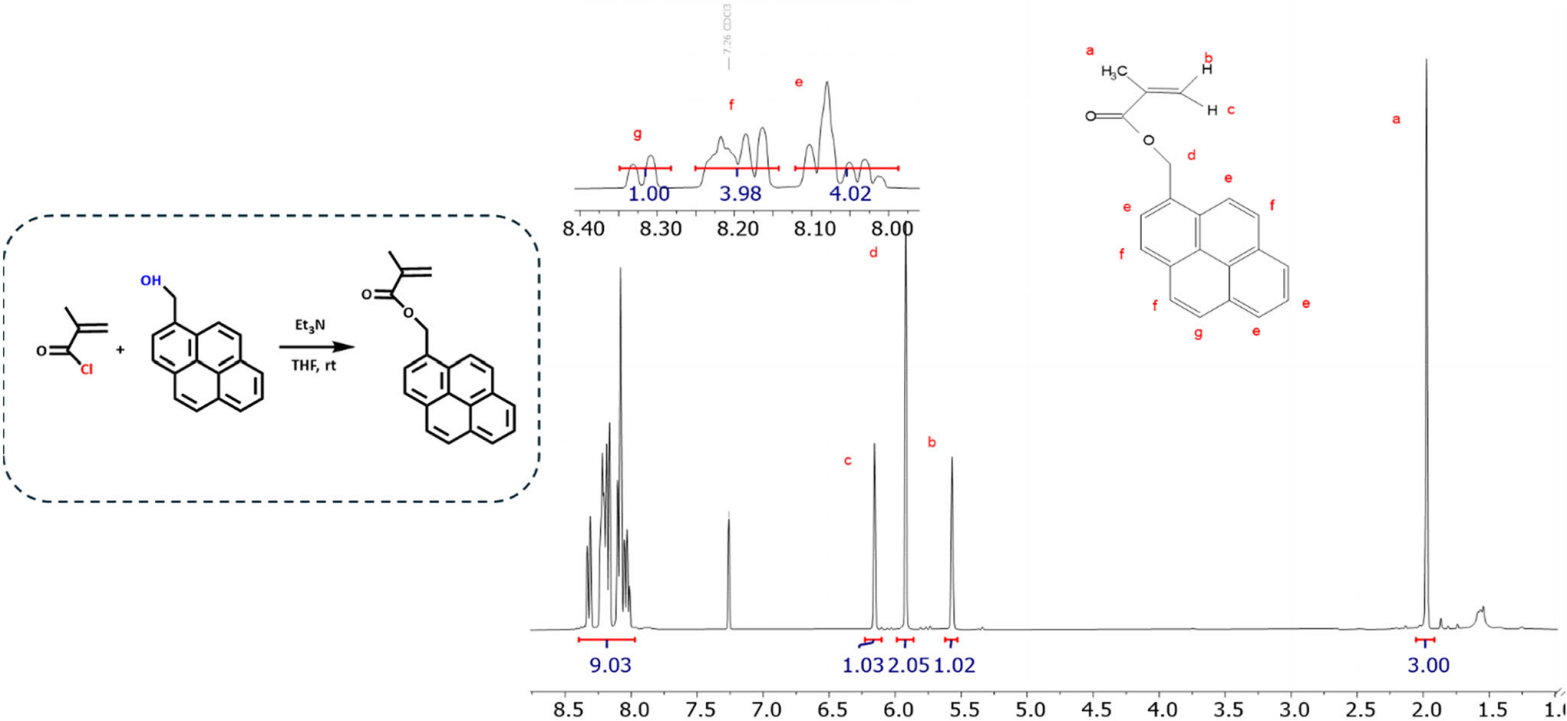
Reaction scheme of 1-pyrenylmethyl methacrylate synthesis and the corresponding ^1^H NMR spectrum in CDCl_3_.

### Polymerization of 1-Pyrenylmethyl Methacrylate via RAFT Polymerization

The synthesized PyMMA monomer was polymerized via the RAFT polymerization using either CPB or CDTPA as the chain transfer agent (CTA), with AIBN as the initiator (Scheme 1). To avoid errors associated with weighing small amounts of reactants, solutions of AIBN and the CTAs were prepared in DMF at known concentrations. Specifically, 25 mg of AIBN were dissolved in 1 mL of DMF, resulting in a concentration of 1.096 × 10^–5^ mol mL ^–1^. Similarly, solutions of 1.19 × 10^–3^ and 1.456 × 10^–5^ mol mL ^–1^ were prepared for CPB and CDTPA, respectively. All stock solutions were prepared in sealed glass vials to prevent solvent evaporation and stored at −20 °C.

**Scheme 1:**
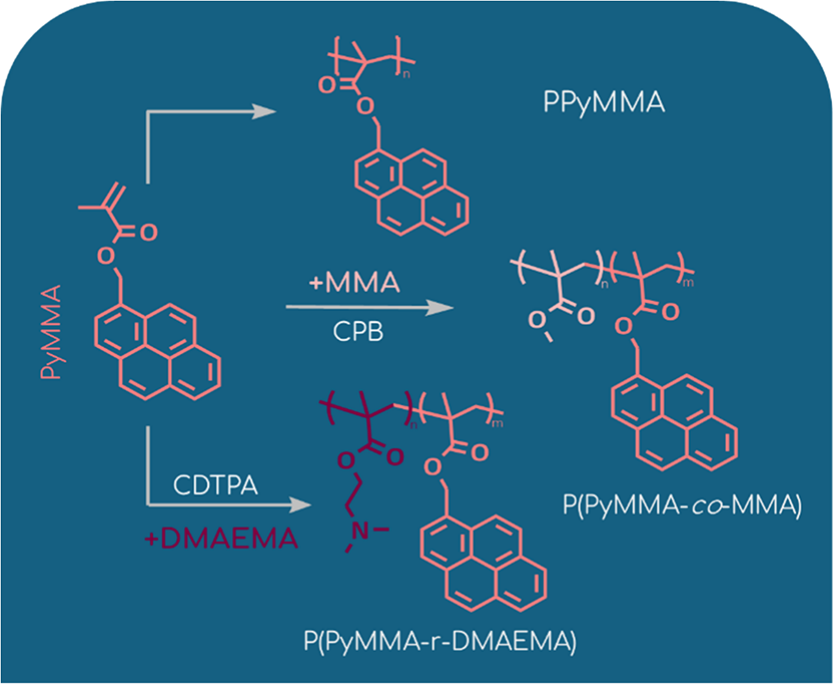
Schematic Representation of the Synthesis Routes for PyMMA Homopolymer and Its Copolymers with MMA and DMAEMA

As a representative example, in a small glass apparatus with a stopcock, 50 mg (0.1 mmol) of PyMMA monomer was combined with 4.2 μL (0.005 mmol) of CTA solution and 45.6 μL (0.0005 mmol) of AIBN solution. DMF was added to bring the total solution volume to 1 mL. The apparatus was then connected to a vacuum line and degassed using freeze–pump–thaw cycles. After being degassed, the apparatus was disconnected from the vacuum line and placed in a 75 °C oil bath. To terminate the polymerization, the apparatus was cooled under running water and then exposed to air.

### Synthesis of PyMMA/MMA and PyMMA/DMAEMA Copolymers

Several samples were synthesized using a method similar to that described for the polymerization of PyMMA. Linear copolymers of P­(PyMMA-*co*-MMA) and a series of P­(PyMMA-*r*-DMAEMA) were prepared (Scheme 1). For the synthesis of the P­(PyMMA-*co*-MMA) copolymer, MMA and PyMMA were introduced into a glass apparatus equipped with a stopcock along with amounts of CTA and AIBN solutions. DMF was employed as the solvent, and the mixture was degassed through the vacuum line. Polymerization was carried out at 75 °C under high vacuum conditions. The resulting samples were diluted with THF, precipitated in hexane, and dried in a vacuum oven.

Additionally, a series of random copolymers of DMAEMA and PyMMA were synthesized by using various feed ratios. A consistent experimental protocol was employed for all of the polymerizations. In each case, a specified molar ratio of the two monomers, along with CTA and AIBN, was transferred to a glass apparatus with a stopcock. DMF was used as the solvent, and the solutions were degassed prior to polymerization, which occurred at 75 °C under high vacuum. The experimental specifications for each synthesis of the random copolymers are presented in Table 2.

## Results and Discussion

### Design, Synthesis, and Characterization

In this study, the design focused on integrating the pyrene chromophore within a methacrylic-based monomer to form a polymerizable unit, facilitating precise incorporation into the polymer backbone through the RAFT polymerization. This approach allowed for controlled integration of pyrene, which can be a challenging task in postpolymerization modifications. The choice of a methacrylate backbone provided strategic advantages: methacrylates are well-suited to various controlled radical polymerization techniques, enabling narrow molecular weight distributions and predictable chain lengths. Additionally, polymethacrylates are generally soluble in a range of organic solvents and exhibit an amorphous morphology, lacking intrinsic crystallinity. In our work, we specifically aimed to preserve the amorphous morphology of the copolymers, as previous studies^22,23,26^ have suggested that such a structure may enhance performance in MALDI-TOF experiments. Incorporating neutral and basic comonomers, specifically MMA and DMAEMA, we explored the effects on the matrix performance of the copolymers. The extended π-conjugation of pyrene was expected to enhance UV absorbance and fluorescence, supporting its application in MALDI-TOF MS. The spectroscopic analyses confirmed the effective incorporation of pyrene, validating both the synthetic strategy and the structural attributes of the material.

### PyMMA Monomer Synthesis and Characterization

For the synthesis of the PyMMA monomer, the protocol utilized is also detailed in a number of studies,^27–29^ with only minor modifications made to the final purification steps. Nuclear magnetic resonance (NMR) spectroscopy was employed to characterize the synthesized monomer. The corresponding peaks in the ^1^H NMR spectrum were consistent with the expected integrations, indicating the high purity of the monomer product, which was free of any triethylamine salts. The observed chemical shifts were as follows: δ ppm 8.28–8.34 (g: −C16H9, pyrene), 8.14–8.24 (f: −C16H9, pyrene) 7.98–8,12 (e: −C16H9, pyrene), 6.10–6.22 (c: =CH2), 5.85–5.98 (d: −O–CH2−), 5.52–5.62 (b: =CH2), 1.9–2.05 (a: −CH3).

### Polymerization of PyMMA via RAFT Polymerization

While PyMMA has been incorporated in various copolymer systems,^30–34^ standalone homopolymers of PyMMA, to the best of our knowledge, have not been studied. Initially, we used 4-cyanopentanoic acid dithiobenzoate (CPB) as a chain transfer agent (CTA) to promote controlled polymerization, a common approach for methacrylate-based monomers. Subsequently, we explored the effect of a trithiocarbonate CTA, cyanomethyl dodecyl trithiocarbonate (CDTPA), to determine if it could provide enhanced control over the polymerization process. In both cases, azobis­(isobutyronitrile) (AIBN) was used as the radical initiator in a 10:1 molar ratio relative to the CTA. All polymerizations were conducted in DMF at 75 °C, the temperature at which AIBN decomposes to produce radicals via homolytic cleavage.

Using CPB, we initially aimed for molecular masses below 10,000 g/mol (samples #1, #2 at Table 1) and obtained homopolymers with low dispersity (*Đ* < 1.2), high yields, and molecular weights close to the theoretically expected ones. When CDTPA was employed to target higher molecular weights, we noted an increase in *Đ* at these elevated values, indicating possible pi-pi stacking interactions among pyrene units at longer chain lengths. This trend is in agreement with known limitations for monomers with π-stacking tendencies, which can further hinder chain extension and solubility beyond certain molecular weights. Nonetheless, as illustrated in the SEC eluograms of samples #3 and #4 (Figure 2), the curves appeared symmetrical, with no indications of side reaction, such as termination, during polymerization.

**Table 1:** Experimental Parameters and Molecular Characteristics of PPyMMA Homopolymers

PPyMMA	mol_PyMMA_/mol_CTA_/mol_AIBN_	$M_{w_{\mathrm{theo}}}$	*M*_w_^α^	*C*_s_ (% w/v)	*Đ*	yield (%)
#1 CPB	166/10/1	9k	7370	25	1.25	72
#2 CPB	332/10/1	6.5k	5880	25	1.18	60
#3 CDTPA	600/10/1	20k	9000	10	1.11	47
#4 CDTPA	1500/10/1	50k	12,000	10	1.21	32

**Figure 2:**
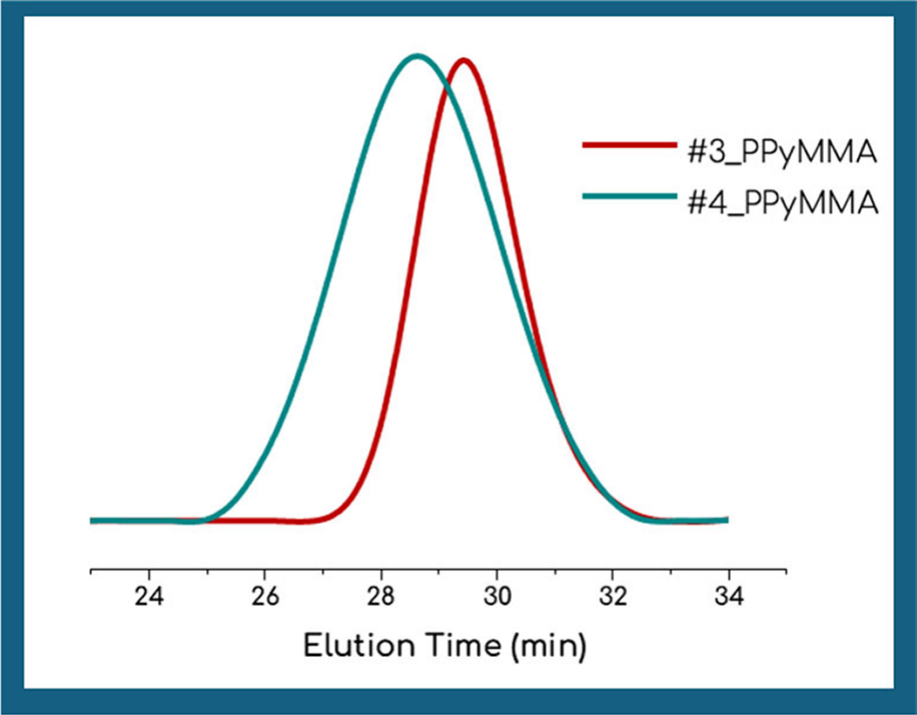
Comparative SEC eluograms of PPyMMA homopolymers (samples #3, #4).

Representative ^1^H NMR spectra for two homopolymers of PyMMA are provided in Figures 3 and 4. In the spectrum for polymer #1 synthesized using CPB, peaks associated with the CTA are absent, making direct molecular weight calculation challenging. However, the peak morphology in the polymer backbone region (0.5–2 ppm) provides insights into tacticity. Here, the absence of the mm (isotactic) peak suggests significant steric hindrance, resulting in predominantly syndiotactic and atactic configurations (rr and mr). In contrast, the spectrum corresponding to the sample synthesized with CDTPA (dark red line), exhibits distinct signals from the chain transfer agent (CTA), as highlighted by the stacked ^1^H NMR spectrum of the pure CTA (green line). This facilitates molecular weight estimation that closely corresponds with the SEC measurements.

**Figure 3:**
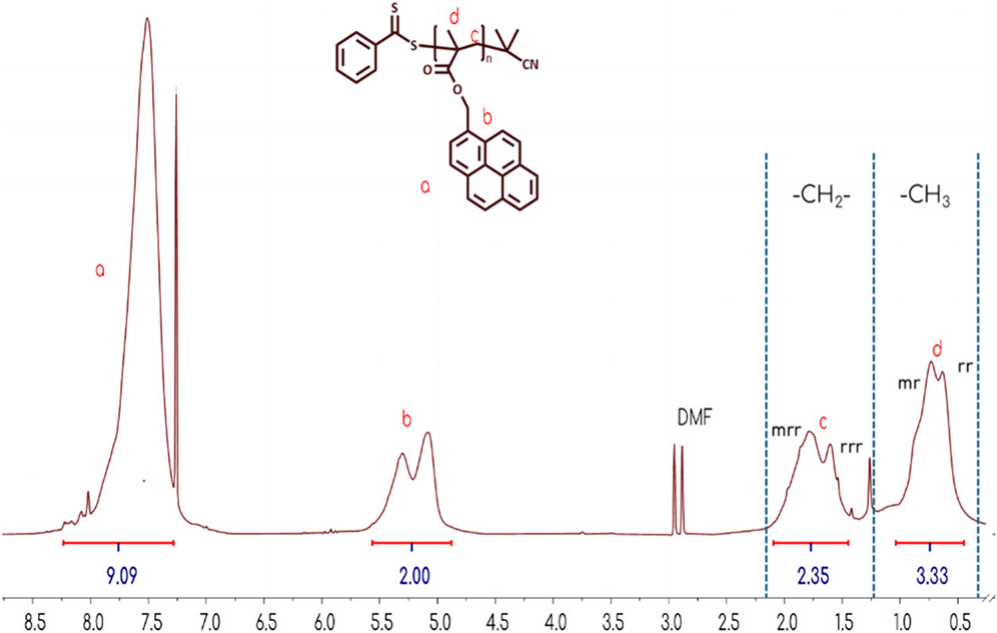
^1^H NMR spectrum of PPyMMA homopolymer using CPB as CTA, with the tacticity highlighted in [0.5–2] ppm.

**Figure 4:**
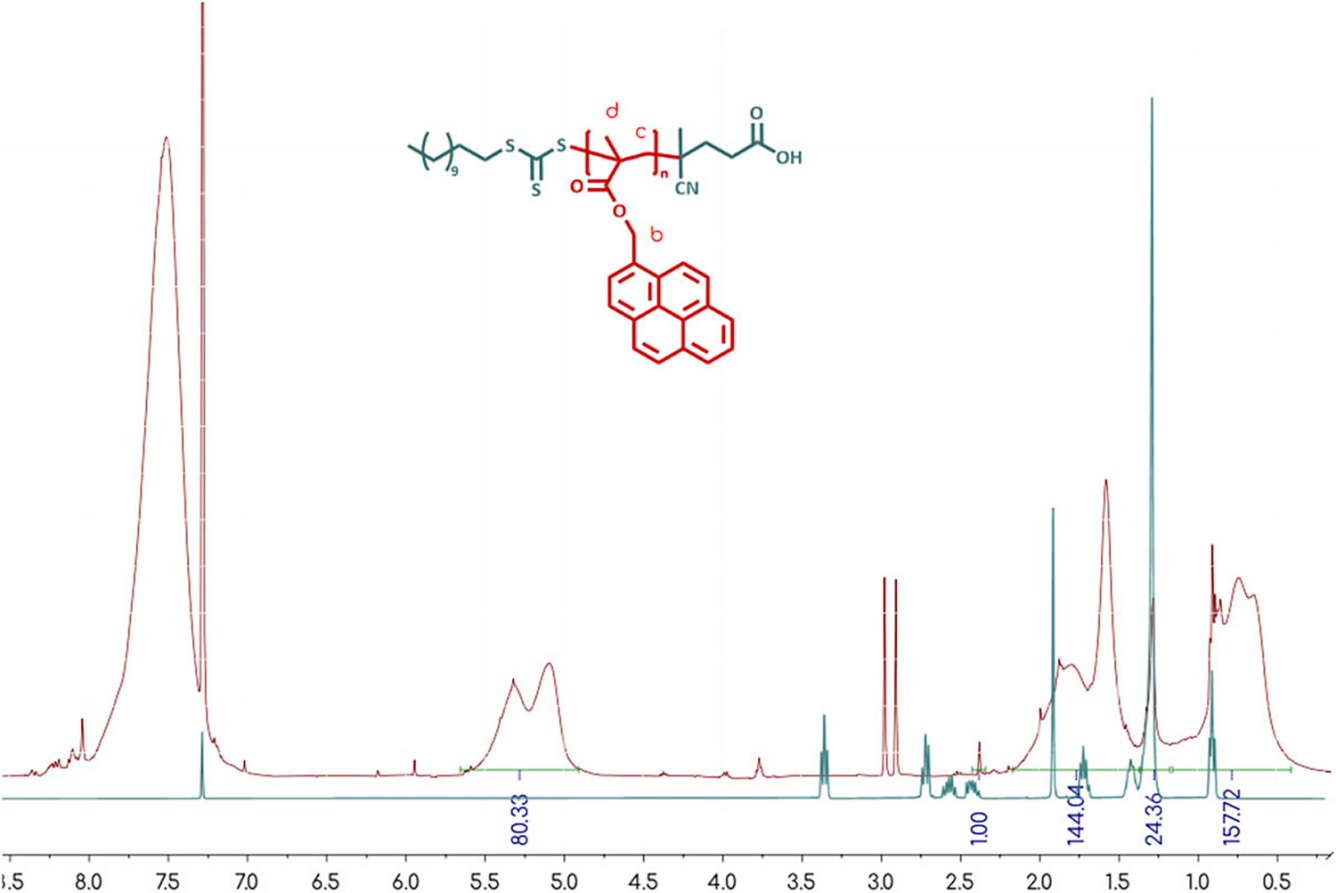
Overlaid ^1^H NMR spectra of the PPyMMA homopolymer using CDTPA as CTA (red line) and pure CDTPA (green line).

### Synthesis of PyMMA/MMA and PyMMA/DMAEMA Copolymers

The RAFT polymerization was employed for the synthesis of random copolymers with MMA with different pyrene weight fractions. To guarantee MMA’s dominance in the copolymer composition, it was always used in excess. CPB served as the chain transfer agent, as reported in prior work from our laboratory,^35^ where it demonstrated excellent control over MMA polymerization. As shown in Table 2, a comparison of the initial monomers’ feed ratio with the resulting polymer composition suggests that PyMMA tends to incorporate more quickly than MMA into the macromolecular chain. This outcome is somewhat unexpected, as the bulkier pyrene unit was anticipated to reduce its reactivity compared to MMA, which lacks a side chain.

**Table 2:** Experimental Parameters and Summarized Results for the P­(PyMMA-*co*-MMA) and P­(PyMMA-*r*-DMAEMA) Copolymers

	feed ratio	molar ratio	mol_M1_/mol_M2_/mol_CTA_/mol_AIBN_	*M*_w_^α^	*Đ*	*C*_s_ (% w/v)	yield %
P(PyMMA_(M1)_-*co*-MMA_(M2)_)	3:1	2.5:1	328/1025/10/1	7.8k	1.25	40	31.3
	6:1	4.5:1	20/120/10/1	11.5k	1.09	50	24.4
	10:1	4:1	152/1520/10/1	11.5k	1.19	50	36.5
P(PyMMA_(M1)_-*r*-PDMAEMA_(M2)_)	20:1	20:1	140/2800/10/1	32k	1.13		50
	10:1	9:1	260/2600/10/1	33k	1.14		49.6

Nevertheless, molecular weights in all samples were close to the targeted values, and dispersities (*Đ*) were consistently low for controlled radical polymerization techniques, as is further supported by the symmetrical appearance of the SEC curves (Figure 5b). Although a higher *Đ* (1.25) was observed in the copolymer with the highest pyrene content, potentially indicating the emergence of pi–pi interactions as a factor contributing to broader molecular weight distributions. ^1^H NMR spectroscopy was used to validate the final copolymers’ compositions by comparing the characteristic peaks for the methylene group of PyMMA (5.0–5.8 ppm) and the methyl group of MMA (3.0–3.7 ppm).

**Figure 5:**
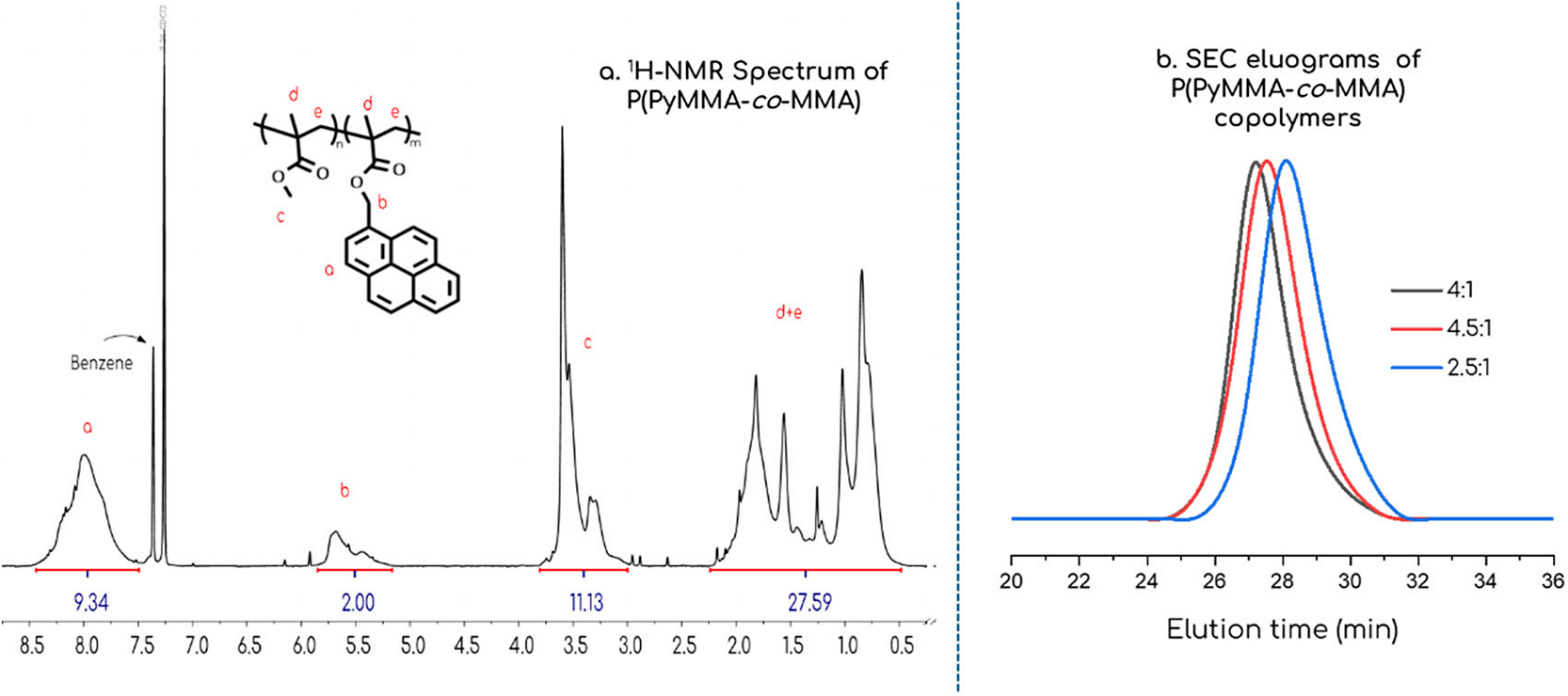
(a) Representative ^1^H NMR spectrum of random copolymer P­(PyMMA-*co*-MMA) [molar ratio, MMA:PyMMA = 4.5:1] and (b) comparative SEC eluograms of P­(PyMMA-*co*-MMA) copolymers with various pyrene content.

In an effort to synthesize polymers with molecular masses exceeding 20 kg/mol, DMAEMA was incorporated alongside the pyrene-based monomer. Following methods reported in the literature^36^ and detailed in the Experimental Section, the RAFT polymerization was performed using CTDPA as the chain transfer agent. Two different molar ratios were prepared, affording polymers with compositions closely matching the intended feed ratios. ^1^H NMR spectroscopy was employed to confirm the consistency of the monomer sequence. By comparison of the peak corresponding to the aromatic protons of the fused pyrene ring system (7.7–8.5 ppm) with that of the two methyl groups (3.8–4.3 ppm) in the DMAEMA segment, the molar ratio of the two monomers in the copolymer was accurately calculated. The corresponding ^1^H NMR spectra are presented in the Supporting Information (Figure S4).

### Reactivity Ratios

To determine whether agreement between feed and final molar ratio is maintained and to evaluate the precise control over the incorporation of both a basic functional group and a chromophore, reactivity ratio calculations were performed. Five feed ratios were selected ([5/1.3/1.1/1.1/3.1/5]), at each polymerization targeting the same theoretical molecular weight and terminated at the same time in order to ensure low conversion. This approach reduced the effect of monomer conversion on the calculation of the reactivity ratios by ensuring a constant monomer composition throughout the reaction time. The results, displayed in Table 3 together with SEC data in Figure 6, were processed using the Fineman–Ross (FR), inverted Fineman–Ross (invFR), and Kelen–Tudos (KT) equations, as well as the COPOINT program.

**Table 3:** Experimental Parameters and Summarized Results for the P­(PyMMA-*r*-DMAEMA) Copolymers

	P(PyMMA-r-DMAEMA)			
feed ratio	molar ratio	*M*_w_ ^a^	*Đ*	yield^b^ %
1:3	1:3	11,300	1.19	14.2
1:1	1:1.3	7300	1.19	28.1
3:1	2.5:1	6800	1.14	20.5
1:5	1:3.5	7500	1.15	50.5
5:1	5:0.5	15,000	1.15	36.1

a Determined via SEC calibrated with PS STDs.

b Determined via gravimetry.

**Figure 6:**
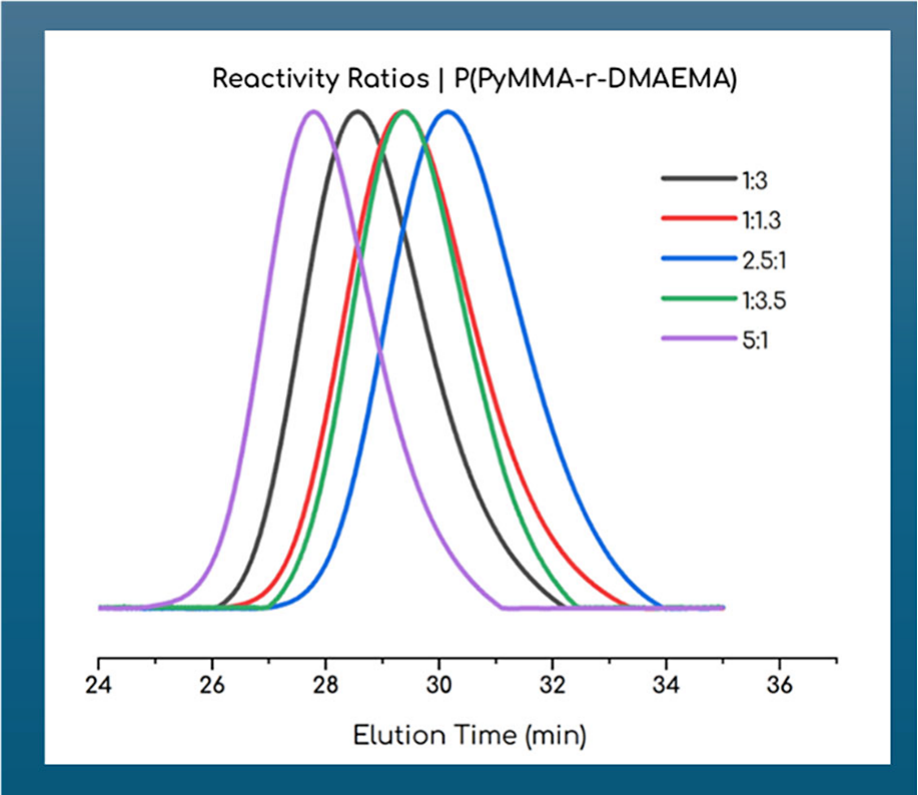
Comparative SEC eluograms of the samples synthesized for reactivity ratio calculation.

As shown in Table 4, calculated reactivity ratios indicate that PyMMA incorporates slightly faster into the copolymer chain than DMAEMA. The dyad composition analysis (Figure 7) shows that as the DMAEMA mole fraction increases, the formation of DMAEMA dyads (M­(A)–M­(A)) rises, while PyMMA dyads (M­(B)–M­(B)) become less frequent. Alternating dyads (M­(A)–M­(B)) peak at a more balanced feed ratio, suggesting optimal conditions for random incorporation. These results imply that adjusting the monomer feed ratio allows for control over copolymer structure, with higher PyMMA content potentially leading to increased pi-pi stacking interactions due to PyMMA clustering that significantly impact the copolymer’s optical properties, solubility, and structural characteristics.

**Table 4:** Reactivity Ratio Calculations Using Fineman–Ross (FR), Inverted Fineman–Ross (invFR), and Kelen-Tudos (KT) Equations and COPOINT Program

	*r*_PyMMA_	*r*_DMAEMA_
Finemann-Ross F-R	0.6454	0.5604
Inv. Finemann-Ross IF-R	0.4807	0.526
Kelen–Tudos K-T	0.7001	0.6115
Ext.Kelen–Tudos.Ext K-T	0.5876	0.485
COPOINT	0.733	0.539

**Figure 7:**
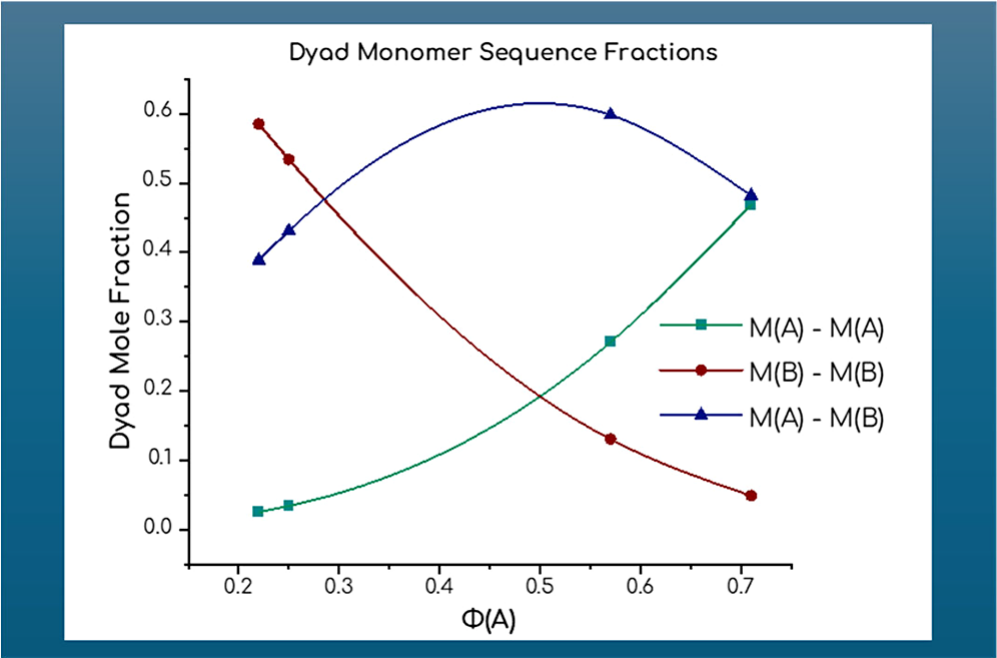
Dyad monomer sequence fractions.

### Optical Properties|Pyrene Content

Pyrene exhibits strong ultraviolet absorption, a unique vibronic fine structure, and outstanding fluorescence characteristics, including separate monomer and excimer emissions. These properties have resulted in a wide variety of applications,^37^ such as light-emitting diode (LED) components,^38^ photoinduced initiators in polymerization,^39^ solubilization agents for single-walled carbon nanotubes (SWNTs)^40^ using pi–pi stacking interactions, and molecular probes to investigate the microenvironments.^41^ Taking account of these properties, we incorporated pyrene into our polymer design as a pendant group to exploit its strong fluorescence and UV absorption features. Several optical characterization methods were utilized to verify that the synthesized polymers possessed such distinctive features.

To quantitatively assess the incorporation of pyrene moieties, we constructed a calibration curve using 1-pyrenylmethyl methacrylate as a reference compound, as shown in Figure 8a. This approach enabled us to calculate the molar extinction coefficient (ε_0_),^42^ by employing the Lambert–Beer law. All solutions were prepared in THF. Notably, 1-pyrenylmethyl methacrylate exhibits an absorption maximum slightly red-shifted (344 nm) compared to parent pyrene (337 nm), as illustrated in Figure 8b. Such a minor bathochromic shift should be attributed to the substitution on the pyrene core, as the methoxymethyl spacer introduces slightly electron-donating effects.

**Figure 8:**
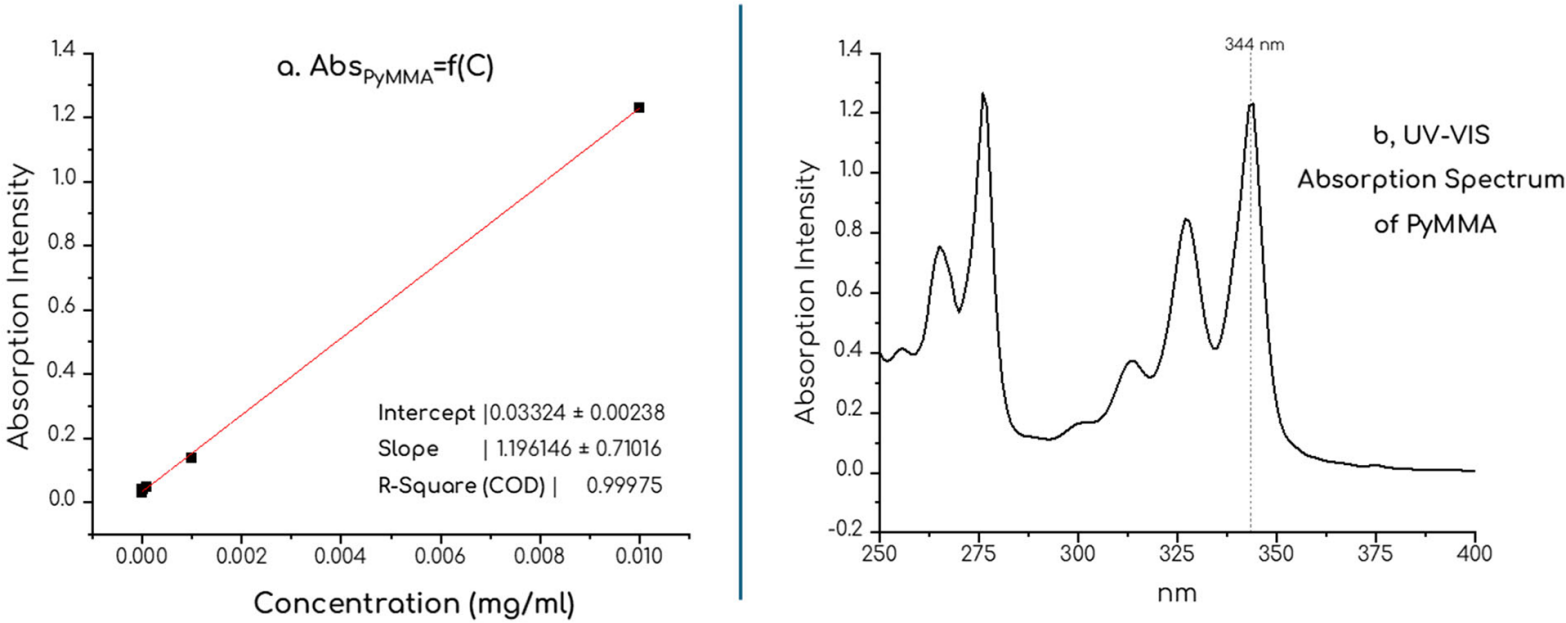
(a) Plot of absorbance vs concentration for PyMMA at 344 nm. The linear relationship confirms the Beer–Lambert law, with the slope corresponding to the molar absorption (ε). Inclusion of 9 points, not visible due to plot scaling. (b) Representative absorption spectrum of PyMMA, *C* = 0.01 mg/mL in THF.

The UV spectra of the synthesized copolymers, as shown in Figure 9a,b, displayed characteristic pyrene absorption bands, confirming that the pendant pyrene groups retained their inherent optical characteristics. The retention of these bands indicates minimal disruption of pyrene’s photophysical properties upon polymerization, supporting its suitability for functional material development.

**Figure 9:**
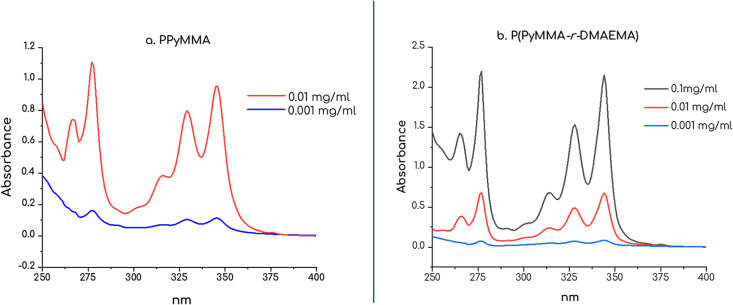
(a) Absorption spectra of homopolymer PPyMMA [12k, DP = 40] in THF and (b) random copolymer P­(PyMMA-*r*-DMAEMA), [33k, molar ratio = 9:1, DP_PyMMA_ = 34] in THF., in comparable concentrations. Note that scaling on the *y*-axis differs.

In the same manner, we initially examined the fluorescence properties of 1-pyrenylmethyl methacrylate. Trial fluorescent measurements on the synthesized polymers suggested strong pi–pi interactions in relatively low concentrations (monomer emission appearing as a characteristic triple band at 379, 397, and 417 nm, and excimer emission centered at 486 nm),^43,44^ so we needed to establish if these kinds of interactions would be present in the free form and/or which is the critical concentration of their emerge. A series of solutions of 1-pyrenylmethyl methacrylate was prepared in the same manner as in the calibration curve mentioned previously. The acquired spectra are provided in the Supporting Information (Figures S5 and S6). From the results obtained, it was observed that only highly concentrated solutions exhibited the characteristic excimer emission band.

Since all synthesized polymers retained the absorption peaks of parent pyrene, we employed the intrinsic fluorescence bands of pyrene to establish a relationship between polymer composition and architecture with the intensity ratio of monomer to excimer emission. The homopolymer exhibited a pronounced excimer emission (Figure 10C), indicating significant pi–pi stacking interactions between adjacent pyrene groups. This suggested a morphology with a high degree of pyrene proximity. In contrast, the random copolymers showed a greater contribution from monomer fluorescence relative to excimer emission.^45,46^ This implies a more spatially distributed arrangement of pyrene units, resulting from the random incorporation of pyrene-functionalized monomers alongside non-pyrene monomers. In-depth analysis regarding *I*_M_/*I*_E_ ratios is beyond the scope of this study; however, it is evident that in the homopolymer structure, excimer emission is almost exclusively evident compared to monomer emission. On the other hand, when comparing the two copolymers, the picture is significantly different. For P­(PyMMA-*r*-DMAEMA) (Figure 10A), the monomer emission intensity is much greater than that of the excimer band. This result supports the random morphology that we previously documented with reactivity ratio calculations. Nonetheless, in P­(PyMMA-*co*-MMA) (Figure 10B), excimer emission seems enhanced compared to the monomer emission. This could be attributed to the higher concentration of the solution but also indicates that pyrene groups are less randomly distributed in the macromolecular chain.

**Figure 10:**
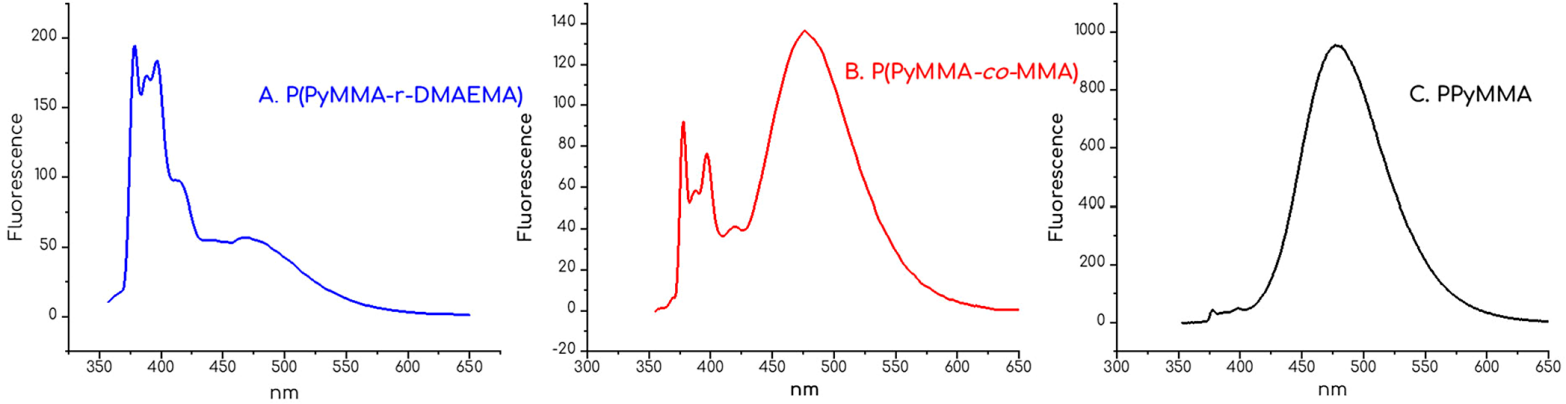
Fluorescence spectra of random copolymers, (A) P­(PyMMA-*r*-DMAEMA) [33k, molar ratio = 9:1], (B) P­(PyMMA-*co*-MMA) [11k, molar ratio = 4:1], and homopolymer (C) PPyMMA [12k], showing emission intensity as a function of wavelength. Excitation wavelength: 344 nm.

### Pyrene-Containing Polymers as MALDI-TOF Matrices

Pyrene, along with other compounds from the PAH family, has already been utilized as a matrix for the analysis of nonpolar macromolecules.^47,48^ Additionally, recent studies^49–51^ have employed pyrene as a label in label-assisted laser desorption ionization (LA-LDI) MS, taking advantage of its well-documented electron transfer capabilities and molecular ion formation. Earlier tests, not presented here, confirmed the optimal matrix concentration and matrix-to-analyte ratio. Given that all pyrene-containing polymers maintain the optical properties of the parent molecule, we initially investigated whether the synthesized polymers would be MALDI-silent, lacking interference peaks in the low molecular weight range (up to 1000 *m*/*z*). Unfortunately, all polymers exhibited two predominant fragment peaks in the 0–1000 *m*/*z* range (with additional ion peaks visible upon spectral magnification, see SI). The first peak, observed at *m*/*z* = 215.0855 (attributed to PyCH^+^), is assigned to a fragment potentially resulting from the cleavage of the ether bond within the pyrene-bearing side chains. A less intense peak appeared at precisely double this *m*/*z* (430.1716), suggesting the presence of an ion potentially originating from a 215.0855 *m*/*z* ion dimer. Although the relative intensity of the alleged dimer-derived fragment ion follows the same trend as the molar pyrene concentration in each polymer and correlates with the trend in excimer formation observed in PL studies, further investigation is required to determine whether the fragment at 430.1716 *m*/*z* results from pre-existing dimers or if they form upon laser ablation. The significant matrix fragmentation, evidenced by the abundant signal at *m*/*z* 215.0855, its dimer, and their contribution to the noise increase in the mass spectra, represent limitations that require attention in future research to enhance overall sensitivity. Exploring alternative designs for the polymeric structures should be considered.

Despite the presence of fragments, it is undeniable that, compared to α-CHCA, a classic organic matrix, interference in the low-mass range is reduced under identical laser intensities (40%). The MALDI-TOFMS spectra of α-CHCA, as a classic organic matrix reference, and the spectra acquired for all pyrene-containing polymers without the presence of analytes or any additives (matrix only) are illustrated in Figure 11. As anticipated, the fragment intensity increases with the pyrene weight fraction in the macromolecules [*n*_pyrene(PPYMMA)_ < *n*_pyrene[P(PyMMA*‑co‑*MMA)]_ < *n*_pyrene[P(PyMMA*‑r‑*DMAEMA)]_], as also evident from the differences in the order of magnitude of the signal intensities on the *y*-axis in Figure 11b across the evaluated polymers.

**Figure 11:**
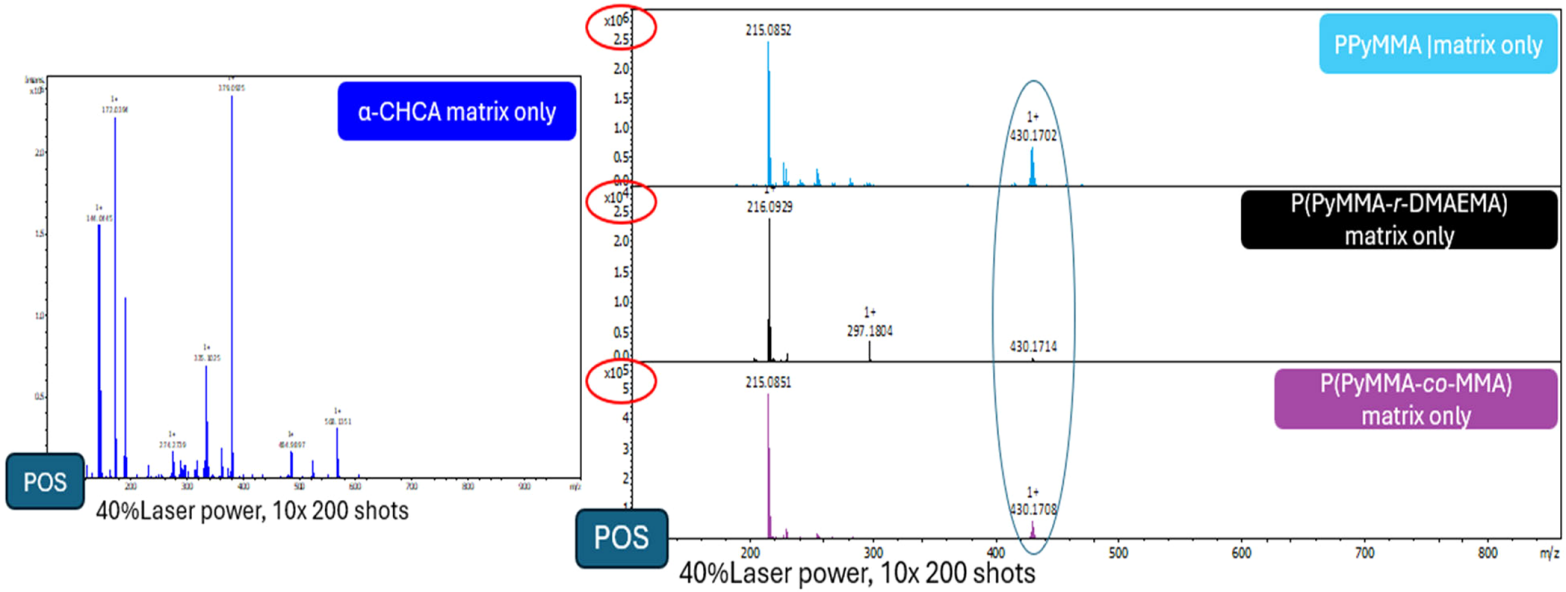
MALDI-TOFMS spectra of α-CHCA (left) and stacked spectra of PPyMMA, P­(PyMMA-*r*-DMAEMA), and P­(PyMMA-*co*-MMA) (right).

Having characterized the fragmentation behavior of the synthesized polymers during MALDI-TOF measurements, we next evaluated their ability to facilitate analyte ionization. To this end, a standard solution mixture of LMWCs of significant environmental concern (SI, Table S1) was prepared. The four tested analytes in this study—trimethoprim, imazapyr, bisoprolol, and sulpiride—were selected not only for their environmental relevance but also because they consist of basic compounds (p*K*_a_ > 7) containing nitrogen atoms with lone pair electrons (e.g., in amine and aromatic nitrogen functionalities). Such sites are highly favorable for protonation, facilitating their detection in positive ion mode. The ionization efficiency of our synthesized polymers was then assessed in comparison to that of P3DDT, a commercially available polymer well-established as an effective matrix for small molecule MALDI-TOF applications.^22,23^ P3DDT thus served as a benchmark for evaluating the ionization efficiency of the novel polymeric matrices.

Analysis of the mass spectra presented in Figure 12 revealed the presence of protonated molecular ions ([M + H]^+^) for all target analytes across the tested polymer matrices. Notably, low-intensity signals corresponding to these analytes were also detected in the absence of a matrix (Figure 12). This observation can be attributed to the inherent structural characteristics of the studied compounds, which, like many small molecules encountered in environmental matrices, possess aromatic rings, potentially capable of absorbing at the laser’s wavelength. Consequently, a degree of matrix-free laser desorption ionization (LDI) can occur, a phenomenon previously reported for other various small molecules in MALDI and LDI-MS analyses.^52,53^ It is evident, however, that when polymers were added as matrices, the signal intensities of the analytes increased substantially for almost all tested polymers (Figures 12 and S7, S8, and S10), proving their efficiency as MALDI matrices, acting as good mediators of laser energy and enhancing analyte ionization.

**Figure 12:**
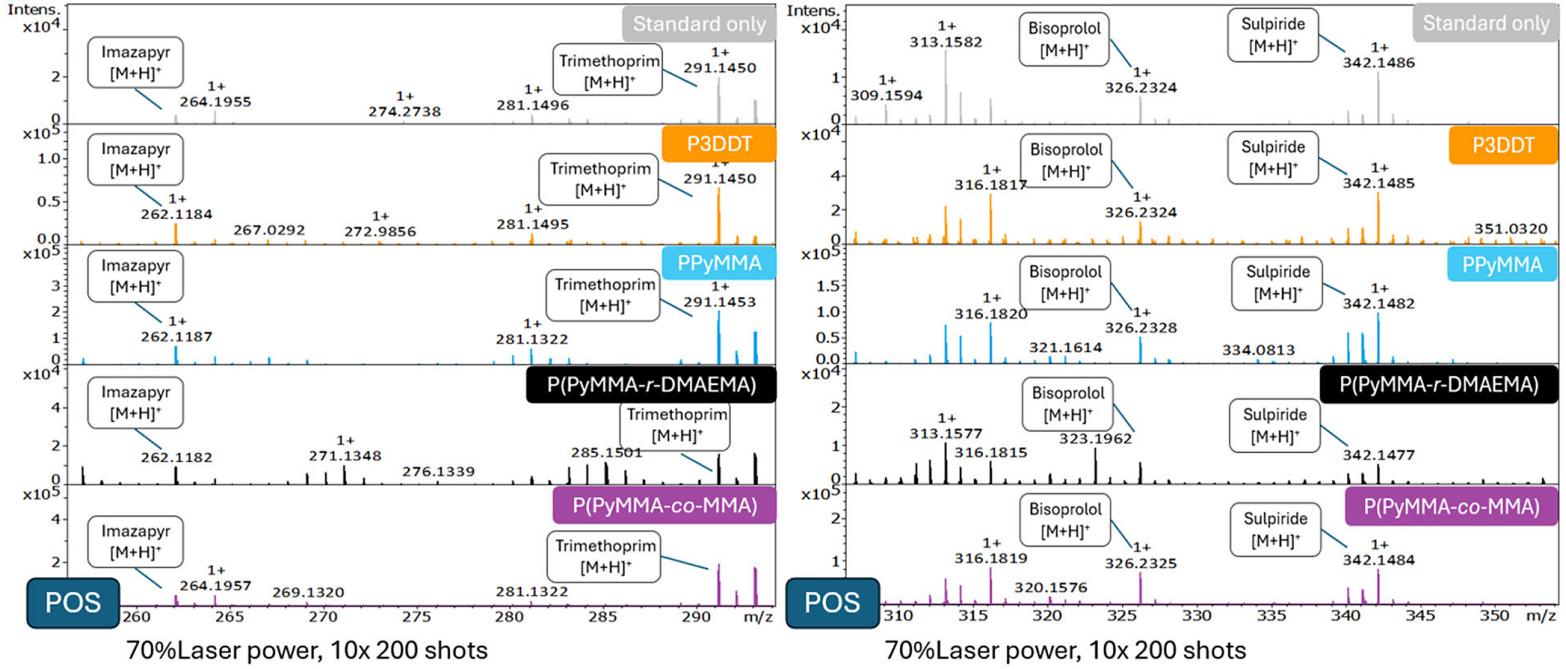
Positive mode MALDI-QTOF-MS spectra of the four evaluated LMWCs without any matrix (light gray) and with P3DDT (orange), PPyMMA (blue), P­(PyMMA-*r*-DMAEMA) (black), and P­(PyMMA-*co*-MMA) (magenta).

More specifically, with PPyMMA, analyte signal intensity increased almost 18 times for imazapyr, almost 10 times for trimethoprim, and almost 9 times for both bisoprolol and sulpiride. For P­(PyMMA-*co*-MMA), analyte signal intensity increased almost 12, 10, 12, and 7 times for imazapyr, trimethoprim, bisoprolol, and sulpiride, respectively. Notably, the ion signal intensities achieved with our synthesized polymers were comparable to those obtained with the commercially available P3DDT (e.g., for imazapyr with P­(PyMMA-*co*-MMA)) or even surpassed those obtained with P3DDT, as was the case for the rest of the analytes for both PPyMMA and P­(PyMMA-*co*-MMA). Overall, PPyMMA demonstrated the highest analyte ionization efficiency among all tested polymers for all four analytes, exhibiting signal intensities greater than those obtained with P3DDT, whereas P­(PyMMA-*r*-DMAEMA) had the lowest analyte signal intensities. It should be noted that the latter exhibited a tendency for slight “spread” on the target plate in the samples, which might have affected the observed results. For clearer visualization and comparative analysis, a bar graph comparing the polymers, standard mixture (STD), and P3DDT is presented in Figure 13, with the underlying data also utilized to generate a heatmap. More detailed depictions of all analyte intensities for each polymeric matrix, in comparison with the spectra containing only the standard analyte and only the matrix, are provided in the Supporting Information. It is pertinent to note that a relatively high laser power (70%) was required for adequate analyte ionization in this study.

**Figure 13:**
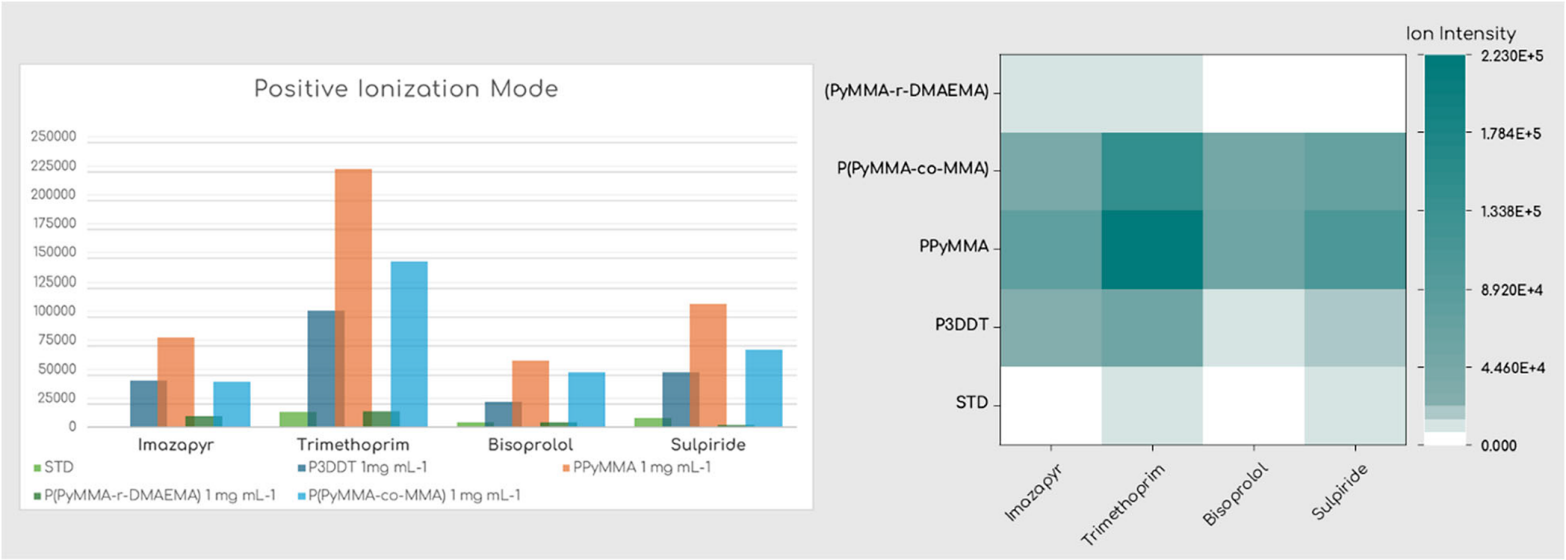
Bar graph (left) showing the ionization efficiencies of the synthesized polymers as matrices and heatmap (right) illustrating analyte signal intensities for various polymers and analytes.

To provide a more comprehensive evaluation of the polymers’ performance as MALDI matrices, signal-to-noise (S/N) ratios for all tested polymers were also compared (SI, Table 2). This analysis revealed that PPyMMA and P­(PyMMA-*co*-MMA) yielded improved S/N values compared with P3DDT, highlighting their potential as efficient alternative polymeric MALDI matrices. Comparison of the S/N values of the polymers with the S/N of the sample containing only the standard was excluded from further evaluation due to the inherently different nature of the tested samples, since they consist primarily of high-purity analytes in a simple hypergrade purity solvent system and are thus expected to exhibit significantly lower background noise. Nevertheless, the ionization efficiency comparisons with the established polymeric matrix P3DDT underscore the promise of these synthesized polymeric structures as alternative MALDI-TOF matrices for the analysis of LMWCs in the positive ion mode.

The pyrene-containing polymers exhibited strong absorbance near the MALDI instrument laser wavelength (Figure 10), suggesting their potential as MALDI matrices. Given pyrene’s excellent optical properties, an electron transfer (ET) pathway might be expected. In typical ET mechanisms, radical cations of neutral analytes are formed and subsequently detected in mass spectra.^48,54,55^ However, the mass spectra obtained using the pyrene-containing polymers predominantly showed the protonated [M + H]^+^ form as the principal ion for all analytes, indicating that analyte protonation was the primary ionization mechanism.

One possible explanation for this observation involves initial photoionization or photoexcitation events, followed by proton transfer reactions. The literature supports proton transfer to analytes from protonated matrix molecules ([Ma+H]^+^), formed by matrix radical cations (Ma^·+^) reactions with neutral matrix molecules after photoionization/photon absorption or protonation from matrix/analyte cluster radical cations via photoionization.^56–59^ Other relevant ionization pathways reported in the literature, among others, include excited-state matrix proton transfer (ESPT) following photoionization or disproportionation reactions.^15^ Nevertheless, the favorability of the proposed photoionization or photoexcitation-initiated and other discussed ionization mechanisms remains uncertain.

The synthesized polymers with higher fragmentation demonstrated higher analyte signal intensities. These observations could be associated with the ionization mechanism. Extended matrix fragmentation could lead to increased matrix-fragment species (e.g., 215. 0855 *m*/*z* [M]^+^, 216.0934 *m*/*z*, potentially the [M + H]^+^, 202.0777 *m*/*z*, a potential pyrene [M]^+^ fragment ion, etc.) (Figure 11 & SI, Figures S11 and S12), which could in turn have acted as the proton transfer agent. The trends of analyte and matrix fragment intensities also align with the pyrene weight fraction in the macromolecules. Nevertheless, the data from our study are insufficient to fully elucidate the exact underlying ionization process­(es), their favorability, and respective contributions. Future investigations are required to further support the proposed ionization mechanisms.

Overall, in this study, three newly synthesized polymers were tested for the first time as potential novel MALDI matrices, with two of the three structures being successfully employed for the detection and identification of imazapyr, trimethoprim, bisoprolol, and sulpiride. Future optimization is needed for circumventing the limitation of intense fragmentation yields of these polymers and increasing the method sensitivity.

## Conclusions

In the race to develop effective MALDI-TOF matrices for the analysis of low-molecular-weight compounds, several approaches have been employed. These include the use of inorganic and hybrid composites, newly synthesized organic compounds, methodologies to suppress background interferences in the low-mass range, and advancements in AI technology.^60^ Polymer science can serve as a useful tool in this process to alleviate drawbacks that originate from conventional SOMs. In this work, we designed and synthesized well-defined polymers in which pyrene is incorporated as a pendant group to serve as a strongly absorbent unit. Homopolymers of 1-pyrenylmethyl methacrylate and copolymers with methyl methacrylate and 2-dimethylamino methacrylate were produced and evaluated for their optical, mechanical, and potential applications in MALDI-TOF experiments. Our results indicate that while significant challenges remain, these materials show notable potential for the analysis of low-molecular-weight organic molecules.

## Supplementary Material



## Data Availability

Data will be made available on request.

## Notes

Additional details on the chemicals used, instrumentation, and experimental settings, as well as ^1^H NMR spectra, DSC measurements, and MALDI-MS results (PDF)

## References

[1] Tanaka K., Waki H., Ido Y., Akita S., Yoshida Y., Yoshida T., Matsuo T. (1988). Protein and Polymer Analyses up to
m/z 100 000 by Laser Ionization Time-of-Flight Mass Spectrometry. Rapid Commun. Mass Spectrom..

[2] Karas M., Glückmann M., Schäfer J. (2000). Ionization in matrix-assisted laser
desorption/ionization: singly charged molecular ions are the lucky
survivors. J. Mass Spectrom..

[3] Karas M., Hillenkamp F. (1988). Laser Desorption
Ionization of Proteins with Molecular
Masses Exceeding 10,000 Da. Anal. Chem..

[4] Karas M., Bachmann D., Hillenkamp F. (1985). Influence
of the Wavelength in High-Irradiance
Ultraviolet Laser Desorption Mass Spectrometry of Organic Molecules. Anal. Chem..

[5] Dourges M.-A., Charleux B., Vairon J.-P., Blais J.-C., Bolbach G., Tabet J.-C. (1999). MALDI-TOF Mass Spectrometry Analysis of TEMPO-Capped
Polystyrene. Macromolecules.

[6] Payne M. E., Grayson S. M. (2018). Characterization
of Synthetic Polymers via Matrix Assisted
Laser Desorption Ionization Time of Flight (MALDI-TOF) Mass Spectrometry. J. Vis. Exp. JoVE.

[7] Pusch W., Kostrzewa M. (2005). Application
of MALDI-TOF Mass Spectrometry in Screening
and Diagnostic Research. Curr. Pharm. Des..

[8] Webster, J. ; Oxley, D. Protein Identification by MALDI-TOF Mass Spectrometry. In Chemical Genomics and Proteomics; Zanders, E. D. , Ed.; Methods in Molecular Biology; Humana Press: Totowa, NJ, 2012; 800, 227–240. 10.1007/978-1-61779-349-3_15 21964792

[9] van
Kampen J. J. A., Burgers P. C., de Groot R., Gruters R. A., Luider T. M. (2011). Biomedical application of MALDI mass spectrometry for
small-molecule analysis. Mass Spectrom. Rev..

[10] Bergman N., Shevchenko D., Bergquist J. (2014). Approaches for the Analysis of Low
Molecular Weight Compounds with Laser Desorption/Ionization Techniques
and Mass Spectrometry. Anal. Bioanal. Chem..

[11] Arnold A., Persike M., Gorka J., Dommett E. J., Zimmermann M., Karas M. (2015). Fast Quantitative Determination of Methylphenidate Levels in Rat
Plasma and Brain Ex Vivo by MALDI-MS/MS. J.
Mass Spectrom..

[12] Li D., Yi J., Han G., Qiao L. (2022). MALDI-TOF Mass Spectrometry in Clinical
Analysis and Research. ACS Meas. Sci. Au.

[13] Leopold J., Popkova Y., Engel K. M., Schiller J. (2018). Recent Developments
of Useful MALDI Matrices for the Mass Spectrometric Characterization
of Lipids. Biomolecules.

[14] Hailat I., Helleur R. J. (2014). Direct Analysis
of Sterols by Derivatization Matrix-Assisted
Laser Desorption/Ionization Time-of-Flight Mass Spectrometry and Tandem
Mass Spectrometry: Analysis of Sterols by MALDI-TOFMS and MALDI-TOF/TOFMS. Rapid Commun. Mass Spectrom..

[15] Zenobi R., Knochenmuss R. (1998). Ion Formation
in MALDI Mass Spectrometry. Mass Spectrom. Rev..

[16] Corinti D., Crestoni M. E., Fornarini S., Pieper M., Niehaus K., Giampà M. (2019). An Integrated
Approach to Study Novel Properties of
a MALDI Matrix (4-Maleicanhydridoproton Sponge) for MS Imaging Analyses. Anal. Bioanal. Chem..

[17] Calvano C. D., Monopoli A., Cataldi T. R. I., Palmisano F. (2018). MALDI Matrices
for Low Molecular Weight Compounds: An Endless Story?. Anal. Bioanal. Chem..

[18] Qiao Z., Lissel F. (2021). MALDI Matrices for
the Analysis of Low Molecular Weight
Compounds: Rational Design, Challenges and Perspectives. Chem. −Asian J..

[19] Abdelhamid H. N. (2016). Ionic Liquids
for Mass Spectrometry: Matrices, Separation and Microextraction. TrAC Trends Anal. Chem..

[20] Porta T., Grivet C., Knochenmuss R., Varesio E., Hopfgartner G. (2011). Alternative
CHCA-Based Matrices for the Analysis of Low Molecular Weight Compounds
by UV-MALDI-Tandem Mass Spectrometry. J. Mass
Spectrom..

[21] Horatz K., Giampà M., Qiao Z., Moestue S. A., Lissel F. (2021). Polymerization
as a Strategy to Improve Small Organic Matrices for Low-Molecular-Weight
Compound Analytics with MALDI MS and MALDI MS Imaging. ACS Appl. Polym. Mater..

[22] Horatz K., Ditte K., Prenveille T., Zhang K., Jehnichen D., Kiriy A., Voit B., Lissel F. (2019). Amorphous Conjugated
Polymers as Efficient Dual-Mode MALDI Matrices for Low-Molecular-Weight
Analytes. ChemPlusChem..

[23] Horatz K., Giampà M., Karpov Y., Sahre K., Bednarz H., Kiriy A., Voit B., Niehaus K., Hadjichristidis N., Michels D. L., Lissel F. (2018). Conjugated Polymers as a New Class
of Dual-Mode Matrices for MALDI Mass Spectrometry and Imaging. J. Am. Chem. Soc..

[24] Chen X.-Y., Wang Y., Ren S.-Y., Li S., Wang Y., Qin K., Li S., Han D.-P., Peng Y., Han T., Gao Z.-X., Gao B.-X. (2022). SI_Amorphous
Poly-N-Vinylcarbazole
Polymer as a Novel Matrix for Determination of Low Molecular Weight
Compounds by MALDI-TOF MS. RSC Adv..

[25] Macha S. F., McCarley T. D., Limbach P. A. (1999). Influence of Ionization Energy on
Charge-Transfer Ionization in Matrix-Assisted Laser Desorption/Ionization
Mass Spectrometry. Anal. Chim. Acta.

[26] Chen X.-Y., Wang Y., Ren S.-Y., Li S., Wang Y., Qin K., Li S., Han D.-P., Peng Y., Han T., Gao Z.-X., Gao B.-X., Zhou H. (2022). Amorphous Poly-N-Vinylcarbazole
Polymer as a Novel Matrix for the Determination of Low Molecular Weight
Compounds by MALDI-TOF MS. RSC Adv..

[27] Wei W., Feng S., Zheng C., Liang G., Zhu F. (2017). Fluorescent
Quantum Yield of Pyrene Probe in Ultrathin Polymer Films. Chin. J. Polym. Sci..

[28] Su C.-H., Wu Z., Lin C.-K. (2019). Polystyrene with Persistently Enhanced Fluorescence:
Photo-Induced Atom Transfer Radical Polymerization Using a Pyrene-Based
Initiator. ChemPhotoChem.

[29] Körpınar B., Saltan F., Akat H., Kınal A. (2020). Synthesis,
Characterization and Computational Investigation of Poly­(Styrene-Co-1-Methylpyrenyl
Methacrylate): Its Efficiency as a Macrophotoinitiator. Int. J. Polym. Anal. Charact..

[30] Jiang J., Tong X., Zhao Y. (2005). A New Design for Light-Breakable
Polymer Micelles. J. Am. Chem. Soc..

[31] You J., Yoon J. A., Kim J., Huang C.-F., Matyjaszewski K., Kim E. (2010). Excimer Emission from
Self-Assembly of Fluorescent Diblock Copolymer
Prepared by Atom Transfer Radical Polymerization. Chem. Mater..

[32] You J., Kim E. (2016). Fluorescent Nanostructures from Aromatic Diblock Copolymers via Atom
Transfer Radical Polymerization. J. Nanosci.
Nanotechnol..

[33] Lou X., Daussin R., Cuenot S., Duwez A.-S., Pagnoulle C., Detrembleur C., Bailly C., Jérôme R. (2004). Synthesis
of Pyrene-Containing Polymers and Noncovalent Sidewall Functionalization
of Multiwalled Carbon Nanotubes. Chem. Mater..

[34] Zhao C., Wu D., Lian X., Zhang Y., Song X., Zhao H. (2010). Amphiphilic
Asymmetric Comb Copolymer with Pendant Pyrene Groups and PNIPAM Side
Chains: Synthesis, Photophysical Properties, and Self-Assembly. J. Phys. Chem. B.

[35] Andreou S., Pantazidis C., Glynos E., Sakellariou G. (2023). Synthesis
of Methacrylate Polyanion Chains via RAFT Polymerization, Kinetic
Study. Thermal Properties of Its Copolymers with MMA and Monomers’
Reactivity Ratios. Eur. Polym. J..

[36] Bagheri A., Boyer C., Lim M. (2019). Synthesis
of Light-Responsive Pyrene-Based
Polymer Nanoparticles via Polymerization-Induced Self-Assembly. Macromol. Rapid Commun..

[37] Figueira-Duarte T. M., Müllen K. (2011). Pyrene-Based
Materials for Organic Electronics. Chem. Rev..

[38] Zhao H., Han W., Bi Y., Xu J., Ma X., Su C. (2024). Pyrene-Modified
Blue Emitters for OLED Applications: Efficiency and Stability Advancements. ChemistrySelect.

[39] Haridharan N., Ramkumar V., Dhamodharan R. (2010). Exploration
of Novel Pyrene Labeled
Amphiphilic Block Copolymers: Synthesis Via ATRP. Characterization and Properties. J. Macromol. Sci. Part A.

[40] Bahun G., Adronov A. (2010). Interactions of Carbon Nanotubes with Pyrene-Functionalized
Linear-Dendritic Hybrid Polymers. J. Polym.
Sci. Part Polym. Chem..

[41] Fu J., Cai Z., Gong Y., O’Reilly S. E., Hao X., Zhao D. (2015). A New Technique
for Determining Critical Micelle Concentrations of Surfactants and
Oil Dispersants via UV Absorbance of Pyrene. Colloids Surf. Physicochem. Eng. Asp..

[42] Ingratta M., Duhamel J. (2007). Correlating Pyrene
Excimer Formation with Polymer Chain
Dynamics in Solution. Possibilities and Limitations.
Macromolecules.

[43] Winnik F. M. (1993). Photophysics
of Preassociated Pyrenes in Aqueous Polymer Solutions and in Other
Organized Media. Chem. Rev..

[44] Haedler A. T., Misslitz H., Buehlmeyer C., Albuquerque R. Q., Köhler A., Schmidt H.-W. (2013). Controlling the
π-Stacking
Behavior of Pyrene Derivatives: Influence of H-Bonding and Steric
Effects in Different States of Aggregation. ChemPhysChem.

[45] Char K., Frank C. W., Gast A. P., Tang W. T. (1987). Hydrophobic Attraction
of Pyrene-End-Labeled Poly­(Ethylene Glycol) in Water and Water-Methanol
Mixtures. Macromolecules.

[46] Siu H., Prazeres T. J. V., Duhamel J., Olesen K., Shay G. (2005). Characterization
of the Aggregates Made by Short Poly­(Ethylene Oxide) Chains Labeled
at One End with Pyrene. Macromolecules.

[47] Macha S. F., Limbach P. A., Savickas P. J. (2000). Application
of Nonpolar Matrices
for the Analysis of Low Molecular Weight Nonpolar Synthetic Polymers
by Matrix-Assisted Laser Desorption/Ionization Time-of-Flight Mass
Spectrometry. J. Am. Soc. Mass Spectrom..

[48] McCarley T. D., McCarley R. L., Limbach P. A. (1998). Electron-Transfer Ionization in Matrix-Assisted
Laser Desorption/Ionization Mass Spectrometry. Anal. Chem..

[49] Yoneda K., Hu Y., Kita M., Kigoshi H. (2016). 6-Amidopyrene as a Label-Assisted
Laser Desorption/Ionization (LA-LDI) Enhancing Tag: Development of
Photoaffinity Pyrene Derivative. Sci. Rep..

[50] Addy P. S., Bhattacharya A., Mandal S. M., Basak A. (2014). Label-Assisted Laser
Desorption/Ionization Mass Spectrometry (LA-LDI-MS): An Emerging Technique
for Rapid Detection of Ubiquitous Cis-1,2-Diol Functionality. RSC Adv..

[51] Hauser J. R., Bergström E. T., Kulak A. N., Warriner S. L., Thomas-Oates J., Bon R. S. (2021). Pyrene Tags for the Detection of Carbohydrates by Label-Assisted
Laser Desorption/Ionisation Mass Spectrometry. ChemBioChem..

[52] Skopikova M., Hashimoto M., Richomme P., Schinkovitz A. (2020). Matrix-Free
Laser Desorption Ionization Mass Spectrometry as an Efficient Tool
for the Rapid Detection of Opiates in Crude Extracts of *Papaver
Somniferum*. J. Agric. Food Chem..

[53] Bronzel J. L., Milagre C. D. F., Milagre H. M. S. (2017). Analysis of Low
Molecular Weight Compounds Using MALDI- and LDI-TOF-MS: Direct Detection
of Active Pharmaceutical Ingredients in Different Formulations. J. Mass Spectrom..

[54] Ramírez-Pradilla J. S., Blanco-Tirado C., Combariza M. Y. (2019). Electron-Transfer Ionization of Nanoparticles,
Polymers, Porphyrins, and Fullerenes Using Synthetically Tunable α-Cyanophenylenevinylenes
as UV MALDI-MS Matrices. ACS Appl. Mater. Interfaces.

[55] Knochenmuss R., Zenobi R. (2003). MALDI Ionization: The Role of In-Plume Processes. Chem. Rev..

[56] Molin L., Seraglia R., Czarnocki Z., Maurin J. K., Pluciński F. A., Traldi P. (2012). On the Primary Ionization Mechanism(s) in Matrix-Assisted
Laser Desorption Ionization. J. Anal. Methods
Chem..

[57] Ehring H., Karas M., Hillenkamp F. (1992). Role of Photoionization
and Photochemistry
in Ionization Processes of Organic Molecules and Relevance for Matrix-Assisted
Laser Desorption Lonization Mass Spectrometry. Org. Mass Spectrom..

[58] Land C. M., Kinsel G. R. (2001). The Mechanism of Matrix to Analyte Proton Transfer
in Clusters of 2,5-Dihydroxybenzoic Acid and the Tripeptide Vpl. J. Am. Soc. Mass Spectrom..

[59] Knochenmuss R. (2004). Photoionization
Pathways and Free Electrons in UV-MALDI. Anal.
Chem..

[60] Padilla C. A., Díaz-Sánchez L. M., Blanco-Tirado C., Combariza A. F., Combariza M. Y. (2024). AI-Guided Design of MALDI Matrices:
Exploring the Electron Transfer Chemical Space for Mass Spectrometric
Analysis of Low-Molecular-Weight Compounds. J. Am. Soc. Mass Spectrom..

