# Supplementary material for: Synthesis and Molecular Characterization of Pyrene-Containing Copolymers as Potential MALDI-TOF MS Matrices
Source: Macromolecules. 2025 Jul 9;58(14):7500–11. doi: 10.1021/acs.macromol.5c00492 (PMC12288060; doi:10.1021/acs.macromol.5c00492)
Supplement: Supplementary file 1 [file ma5c00492_si_001.pdf]

Supporting Information

"Synthesis and Molecular Characterization of Pyrene-Containing Copolymers as Potential MALDI-TOF MS Matrices"

Marileta Tsakanika,[1†] Eleni Aleiferi,[2†] Dimitrios Damalas,[2] Anastasia Stergiou,[1] Nikolaos S. Thomaidis,[2] Georgios Sakellariou.[1*]

[1] Laboratory of Industrial Chemistry, Department of Chemistry, National and Kapodistrian University of Athens, Greece, Panepistimiopolis, Zografou, 15771, Athens, Greece.

[2] Laboratory of Analytical Chemistry, Department of Chemistry, National and Kapodistrian University of Athens, Greece, Panepistimiopolis, Zografou, 15771, Athens, Greece.

Materials & Methods

For the monomer synthesis, 1-pyrenemethanol (TCI Chemicals) and methacryloyl chloride (Sigma-Aldrich) were used as received. The chain transfer agents (CTAs) for the polymerizations, specifically 2-cyano-2-propyl benzodithioate (CPB) (Sigma-Aldrich) and 4-cyano-4-[(dodecylsulfanylthiocarbonyl)sulfanyl]pentanoic acid (CDTPA) ((TCI Chemicals), were also used without further purification. Triethylamine (Merck), tetrahydrofuran (Merck), benzene (Merck) and dimethylformamide (CARLO ERBA) were dried, distilled and kept under vacuum for future use. AIBN (Sigma-Aldrich) was recrystallized from methanol twice and stored at 3 ^{o}C. Methyl methacrylate, MMA (Sigma-Aldrich) and 2-dimethylaminoethyl methacrylate, DMAEMA (Sigma Aldrich) were treated with CaH_2 overnight, distilled and kept under vacuum to be used in a maximum time span of 7 days. For MALDI-TOF MS analysis, tetrahydrofuran and was purchased from Fluka (Buchs, Switzerland) in HPLC grade. Acetonitrile and methanol (MeOH) were hyper grade for LC-MS from Merck (Darmstadt, Germany). Trifluoroacetic acid (TFA) was purchased in HPLC grade from Sigma-Aldrich (Stenheim, Germany). Distilled water was provided by a Milli-Q purification apparatus (Millipore Direct-Q UV, Bedford, MA, USA). LMWC analytes were commercially available and purchased in analytical standard grade. Analyte standards of Trimethoprim, Bisoprolol, Sulpiride and Imazapyr were prepared in separate stock solutions at a concentration of 1000 μg mL^{-1} in MeOH and stored at -20 ^{o}C. A mixture solution containing multiple standard analytes, was prepared in a glass vial, at a final concentration of approximately

10 μg mL^{-1} for each analyte and stored at -20 ^oC. For the scope of this study, only four of the analytes of the mixture, namely, Trimethoprim, Imazapyr, Bisoprolol and Sulpiride were employed for the evaluation of ionization efficiency of the polymers. α-Cyano-4-hydroxycinnamic acid (α-CHCA) and regiorandom Poly(3-dodecylthiophene-2,5-diyl) (P3DDT) were procured from Sigma Aldrich (Stenheim, Germany)

Size exclusion chromatography (SEC) was employed to determine the number-average molecular weight (M_n) and the molecular weight distribution values, represented as $Đ = M_w/M_n$. The analysis was conducted using a system comprised of a Waters 600 high-performance liquid chromatographic pump, Waters Ultrastyragel columns (HR2, HR4, HR3, and HR4E), and a Waters 410 differential refractometer. A chloroform ($CHCl_3$) solution containing 2% triethylamine was utilized as the eluent at a flow rate of 1 mL min^{-1}, with the system maintained at 42 °C. Polystyrene samples were used as calibration standards. FTIR measurements were performed with a Perkin Elmer Spectrum One instrument, in KBr pellets at room temperature, in the range of 450–4000 cm^{-1}. *NMR measurements* were carried out on a 400 MHz Bruker Avance Neo instrument, using $CDCl_3$ as a solvent at 298 K.

Thermogravimetric analysis was performed with a TGA Q50 (TA Instruments), using a platinum pan as a sample holder. The measurements were carried out with a 10 ^oC/min heating rate under N_2 atmosphere. *Differential scanning calorimetry (DSC)* measurements, were performed employing a TA Q200 DSC apparatus (TA Instruments, USA), calibrated with sapphires for heat capacity and indium for temperature and enthalpy, on samples of ~5-11 mg in mass closed in Aluminum TZero pans (TA), in the temperature range from 20 to 220 ^oC in nitrogen atmosphere of high purity (99.9995%). In the first heating scan from RT to 180 or 220 ^oC at 10 ^oC/min the thermal contact between the sample and the pan is optimized, whereas any thermal history is erased, and any remaining humidity / solvents are evaporated (scan 1). Then, (scan 2) the melted samples were cooled to 20 ^oC at 10 ^oC/min and, subsequently, heated to the maximum temperature at 10 ^oC/min.

Ultraviolet-visible (UV-Vis) *measurements* were conducted using a Lambda 650 UV-Vis spectrometer (PerkinElmer). The analyses were performed in quartz cuvettes with a path length of 1 cm, obtained from Starna Scientific Ltd. Prior to measurements, the instrument was calibrated to ensure accuracy. Absorbance spectra were recorded over a wavelength range of 250 to 800 nm to evaluate the optical properties of the samples. For the *fluorescence study*, a

Shimadzu RF-5301 fluorometer was employed, which is equipped with a double monochromator for both excitation and emission, minimizing stray light and enhancing the resolution of spectral data. Measurements were conducted at room temperature.

MALDI mass spectrometry (MS) spectra were acquired by the laboratory of Analytical Chemistry, Department of Chemistry, National and Kapodistrian University of Athens (N.K.U.A), using a Bruker Trapped Ion Mobility Spectrometry timsTOF fleX mass spectrometer (Bruker Daltonics, Bremen, Germany), equipped with a SmartBeam III Nd:YAG laser (355 nm), operating in the MALDI mode with the TIMS mode off. For all measurements, layers of the matrix and analyte were applied on an MTP 384 ground steel target plate (Bruker Daltonics, Bremen, Germany), using a matrix first drop-coating technique derived from the dried droplet method. The optimal layer thickness for each matrix was qualitatively assessed by evaluating various combinations of analyte and matrix concentrations in preliminary tests. Regarding the polymeric matrices, 1 mg mL^{-1} provided optimum results hence this concentration level was used for the ionization efficiency comparisons. Standard analyte solution concentration remained constant at 10 μg mL^{-1} for all measurements. A 10 mg mL^{-1} solution of α-CHCA, was prepared in 1 mL of 0.1% TFA in water and acetonitrile at a 70:30 ratio (TA30), sonicated for 10 min at ~25-30 °C and then centrifuged in a NEYA 16R centrifuge at 9000 rpm, for 10 min at 25 °C. Matrix solutions of the polymers were prepared at a final concentration of 10 mg mL^{-1} in 2 mL Eppendorf tubes, in THF. Then, dilution was performed to form the final matrix solutions of 1 mg mL^{-1}. For the samples containing only the matrices, 0.6 μL of each matrix solution were deposited on the target plate and left to dry at room air for ~ 2min ("matrix only", n=1 replicate). For the polymer samples containing both the matrix and the standard analytes, 0.6 μL of the matrix solution of 1 mg mL^{-1} were deposited separately on the plate and left to dry for ~2 min. Then, 0.6 μL of the analyte standard solution mixture was deposited on top of the already deposited matrix and left to dry for another ~2 min as well (n=3 replicates). 0.6 μL of only the standard solution sample were also deposited on the plate (n=7 replicates). Regiorandom Poly(3-dodecylthiophene-2,5-diyl) (P3DDT) from Sigma Aldrich (Stenheim, Germany) was used for comparison purposes with the results of our newly synthesized polymers.

Measurements were performed in the positive ionization mode. Prior to analysis a 10mM sodium formate solution was used for external mass calibration of the instrument. Each sample spectra consisted of a total of 2,000 shots, compiled from 10 bursts of 200 shots each, with 70% laser

power, unless stated otherwise in the text or figures. MALDI plate offset was at 50 V, deflection 1 delta was set at 70.0V, funnel 1 RF at 250.0 Vpp, funnel 2 RF at 200.0 Vpp and multiple RF at 200.0 Vpp. Collision energy was set at 10.0 eV and collision RF at 850.0 Vpp. Transfer time was 60.0 μs, pre pulse storage 5.0 μs, and ion energy was set at 5.0 eV. Scan range region was 50 – 1000 m/z. DataAnalysis 6.1 by Bruker Daltonics, was used for data treatment. For the identification workflow, intensity thresholds, mass accuracy and isotopic pattern fit of each analyte were used as identification criteria. An intensity threshold of 1000 was set for all target analytes, and all peaks were optically observed. For mass accuracy, a narrow limit of ±2 mDa difference between the theoretical and the experimental mass (Δm/z) was chosen, and for isotopic pattern fitness, mSigma values ranging between 20 and 100 indicated a good correlation between the theoretical and the experimental isotopic pattern. Lastly, the spectra of the detected analytes with each polymer matrix solution sample were compared to the spectra of the sample containing only analyte solution and the matrix solution sample, respectively.

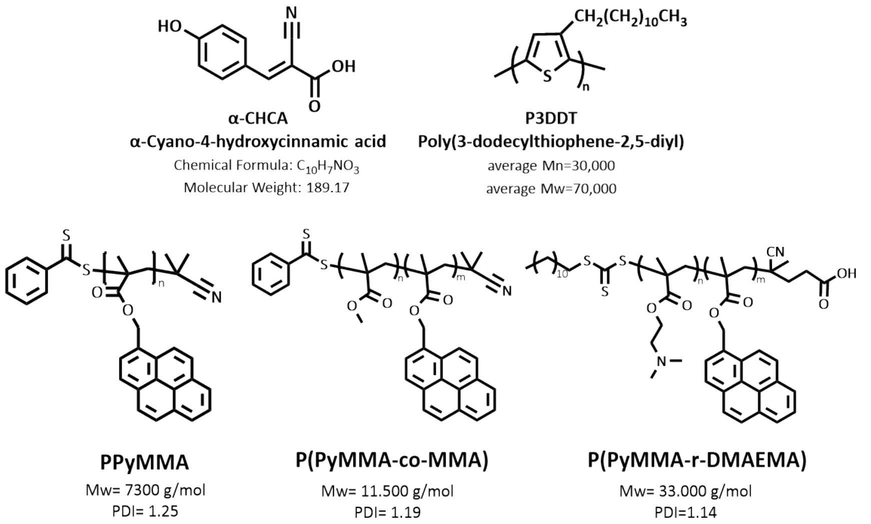

Figure S1 Chemical Structures of α-CHCA (α-Cyano-4-hydroxycinnamic acid), P3DDT [Poly(3-dodecylthiophene-2,5-diyl)] & the synthesized polymers in this study, PPyMMA, P(PyMMA-co-MMA) and P(PyMMA-r-DMAEMA) with CTA end groups shown.

Table 1 Structures, Chemical Formulas of the four Analytes evaluated in POSITIVE detection mode

	Structure	Name & Chemical Formula	Exact monoisotopic mass (mDa)	Theoretical $[M+H]^+$ Ion Mass (m/z)	Substance Category
DETECTION MODE | POSITIVE	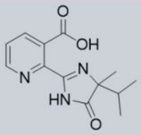	**Imazapyr** $C_{13}H_{15}N_3O_3$	261.1113	262.118	Plant Protection Products, herbicides
	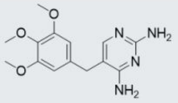	**Trimethoprim** $C_{14}H_{18}N_4O_3$	290.1379	291.1452	Pharmaceuticals antibiotics
	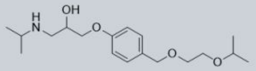	**Bisoprolol** $C_{18}H_{31}NO_4$	325.2253	326.2326	Pharmaceuticals beta blockers
	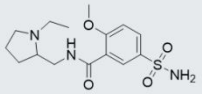	**Sulpride** $C_{15}H_{23}N_3O_4S$	341.1409	342.1482	Pharmaceuticals antidepressants & antipsychotic drugs

Additional ^{1}H-NMR Spectra

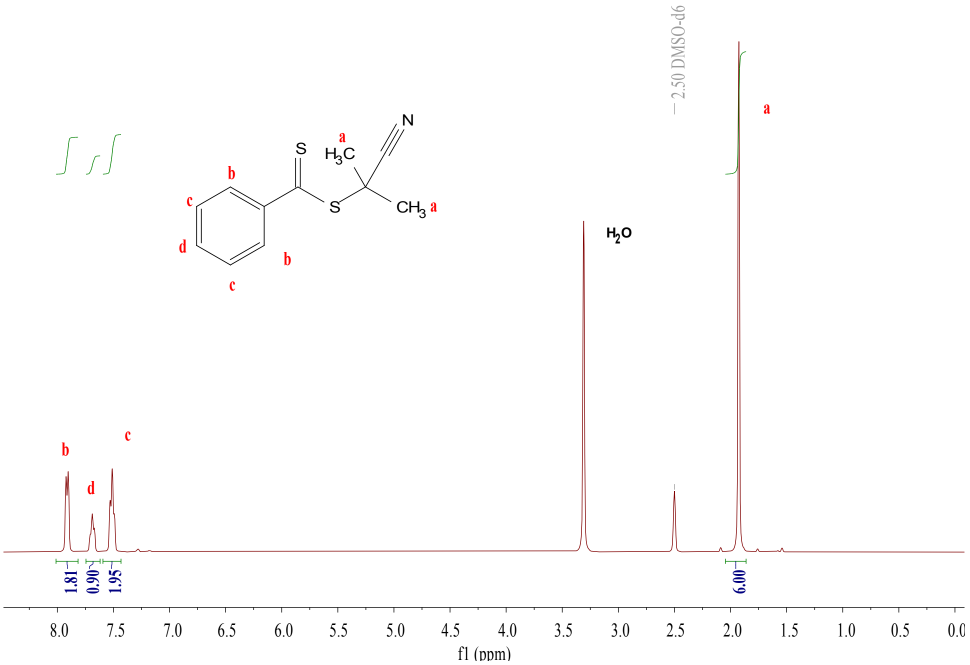

Figure S2 ^{1}H-NMR spectrum of 2-cyano-2-propyl benzodithioate (CPB) in DMSO-d6

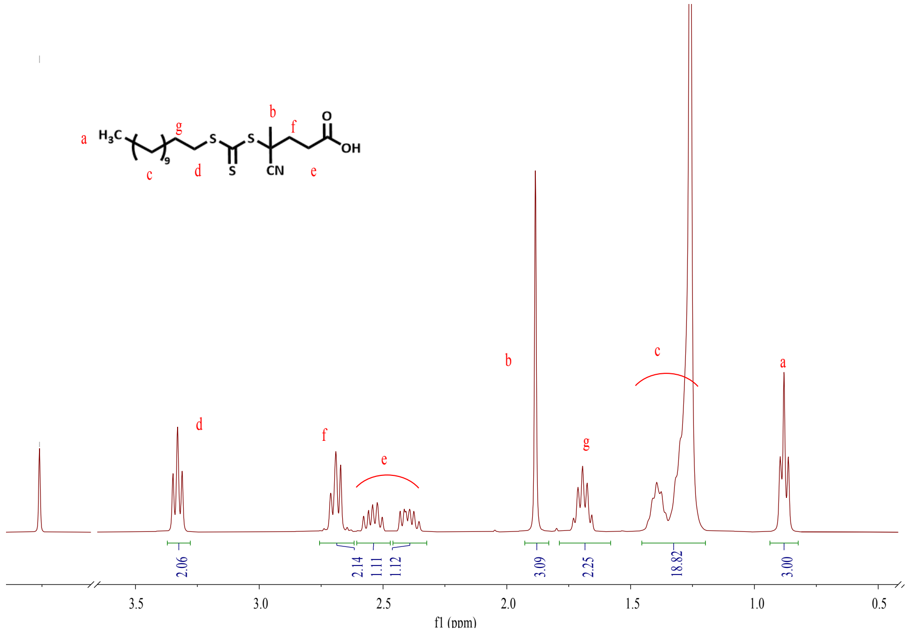

Figure S3 ^{1}H-NMR spectrum of 4-cyano-4-[(dodecylsulfanylthiocarbonyl)sulfanyl]pentanoic acid (CDTPA) in $CDCl_3$

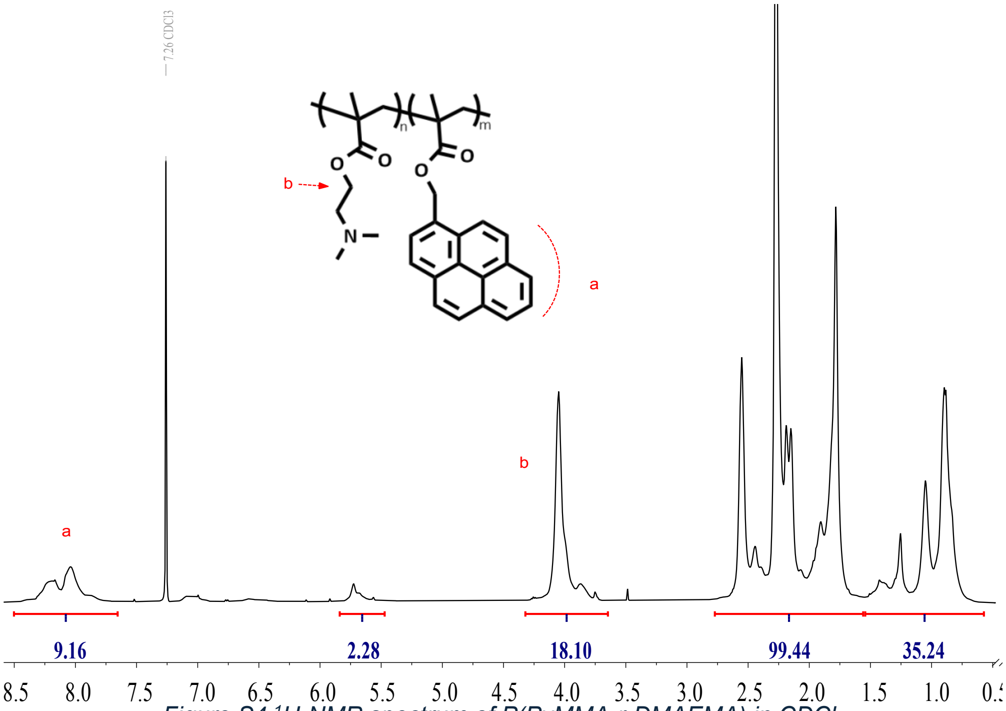

Figure S4 ^{1}H-NMR spectrum of P(PyMMA-r-DMAEMA) in $CDCl_3$

UV-Vis & Fluorescence Measurements

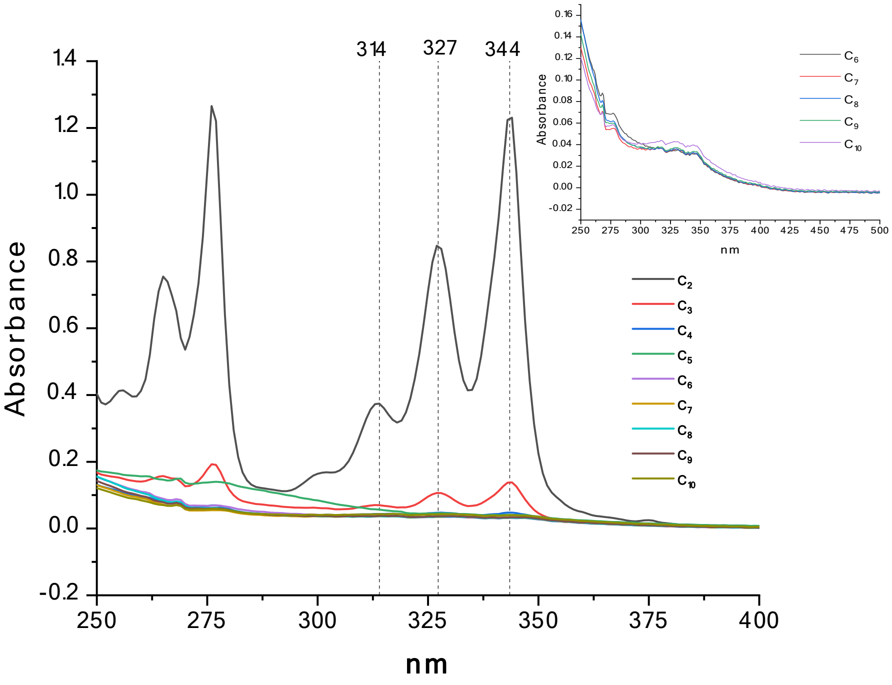

Figure S5 Overlaid UV-vis spectra of PyMMA in concentrations ranging from C_2-C_{10}= 10^{-2}-10^{10} mg ml^{-1}

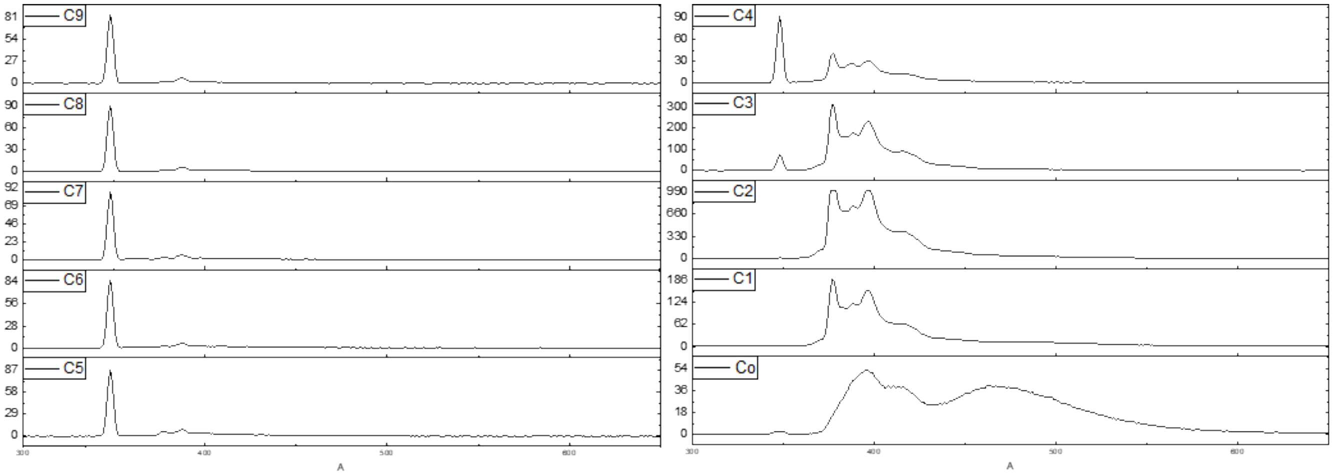

Figure S6 Stacked Fluorescence spectra of PyMMA in concentrations ranging from C_0-C_{10}= 1-10^{10} mg ml^{-1}

MALDI-TIMS-QTOF Spectra

In this section of the supporting information the MALDI MS spectra of all polymers tested in this study are presented in more detail. For each polymer, the spectra containing only the standard solution mixture (upper spectra) and the spectra containing only the matrix (middle spectra) are compared with the spectra of each sample containing both the polymer and the standard solution mixture (lower spectra). Analyte ionization with the addition of polymers was enhanced when polymers were added as matrices (Figures S7, S8, S10) with the only exception the polymer P(PyMMA-*r*-PDMAEMA) (Figure S9).

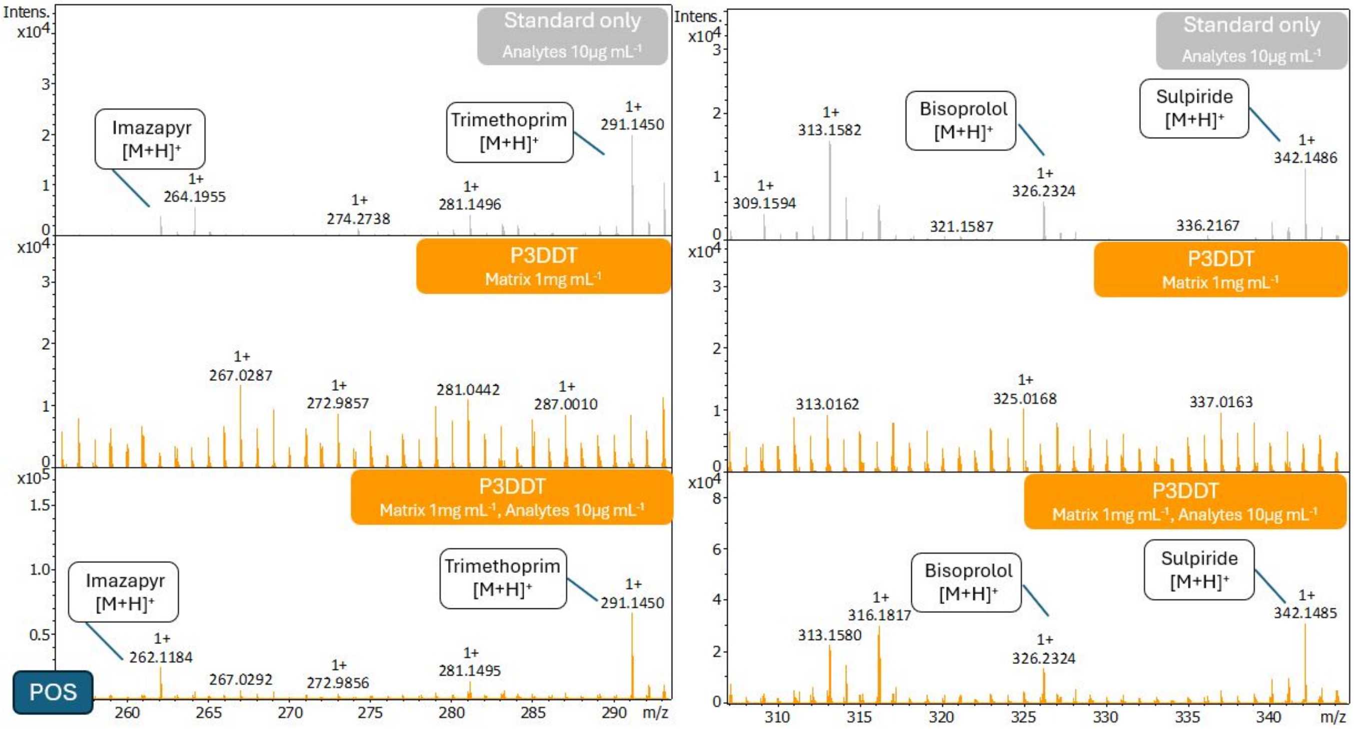

Figure S7 Positive mode MALDI-QTOF-MS spectra of Imazapyr, Trimethoprim, Bisoprolol, and Sulpiride acquired using P3DDT (orange, lower spectra) as matrix, along with spectra obtained without any matrix (light grey, upper spectra) and spectra containing only the matrix (orange, middle spectra).

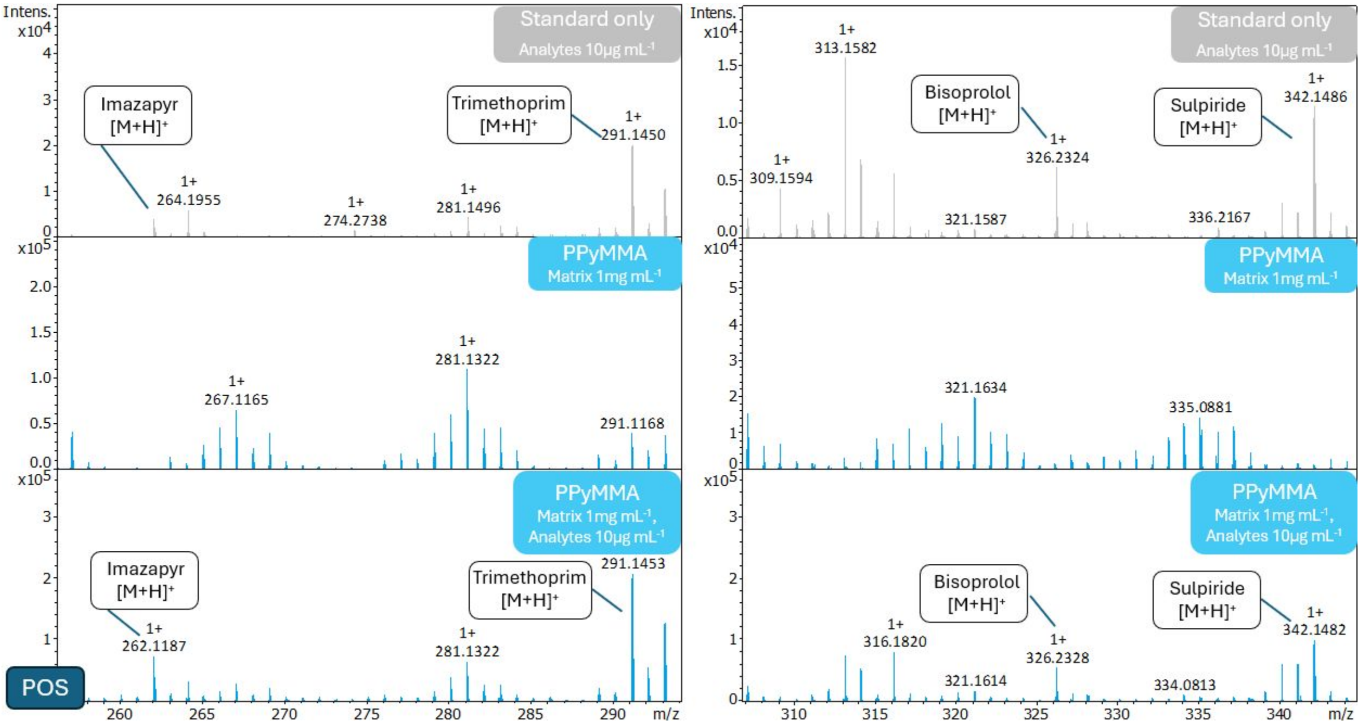

Figure S8 Positive mode MALDI-QTOF-MS spectra of Imazapyr, Trimethoprim, Bisoprolol, and Sulpiride acquired using PPyMMA (blue, lower spectra) as matrix, along with spectra obtained without any matrix (light grey, upper spectra) and spectra containing only the matrix (blue, middle spectra).

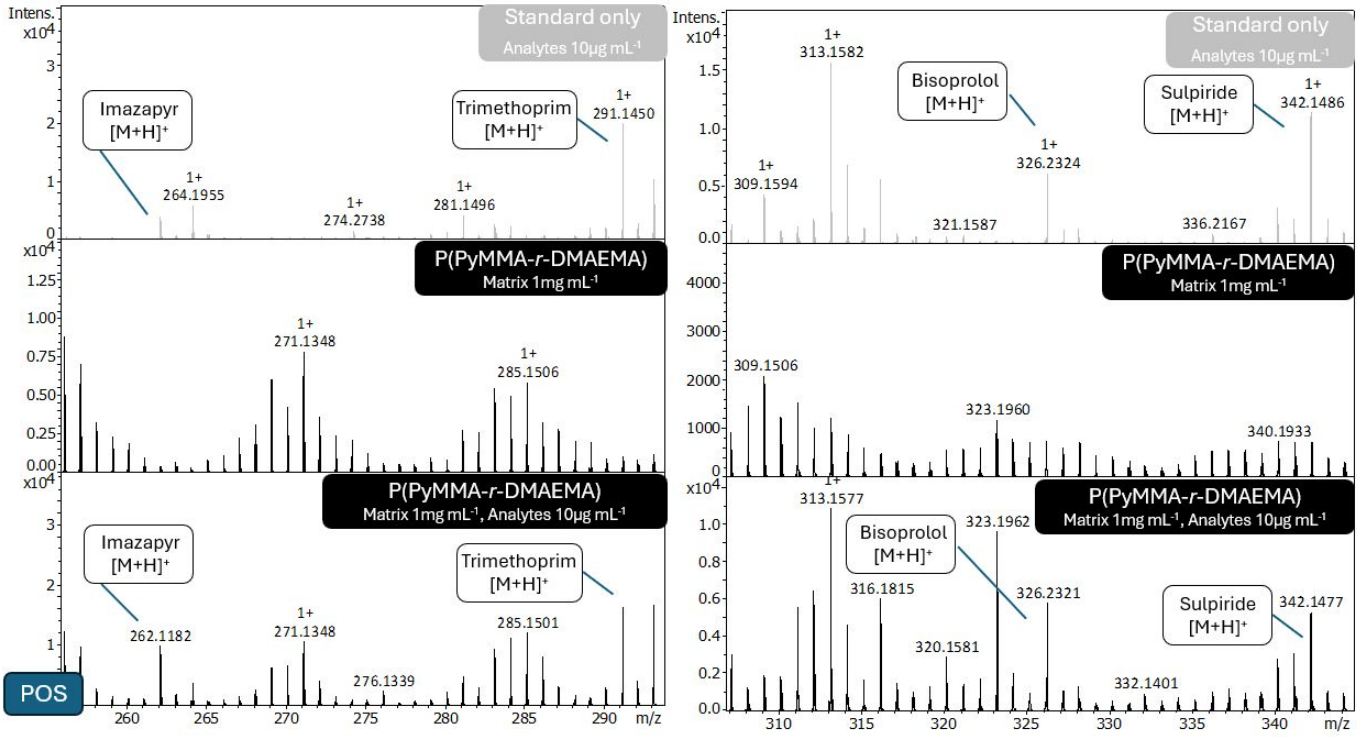

Figure S9 Positive mode MALDI-QTOF-MS spectra of Imazapyr, Trimethoprim, Bisoprolol, and Sulpiride acquired using P(PyMMA-r-DMAEMA) (black, lower spectra) as matrix, along with spectra obtained without any matrix (light grey, upper spectra) and spectra containing only the matrix (black, middle spectra).

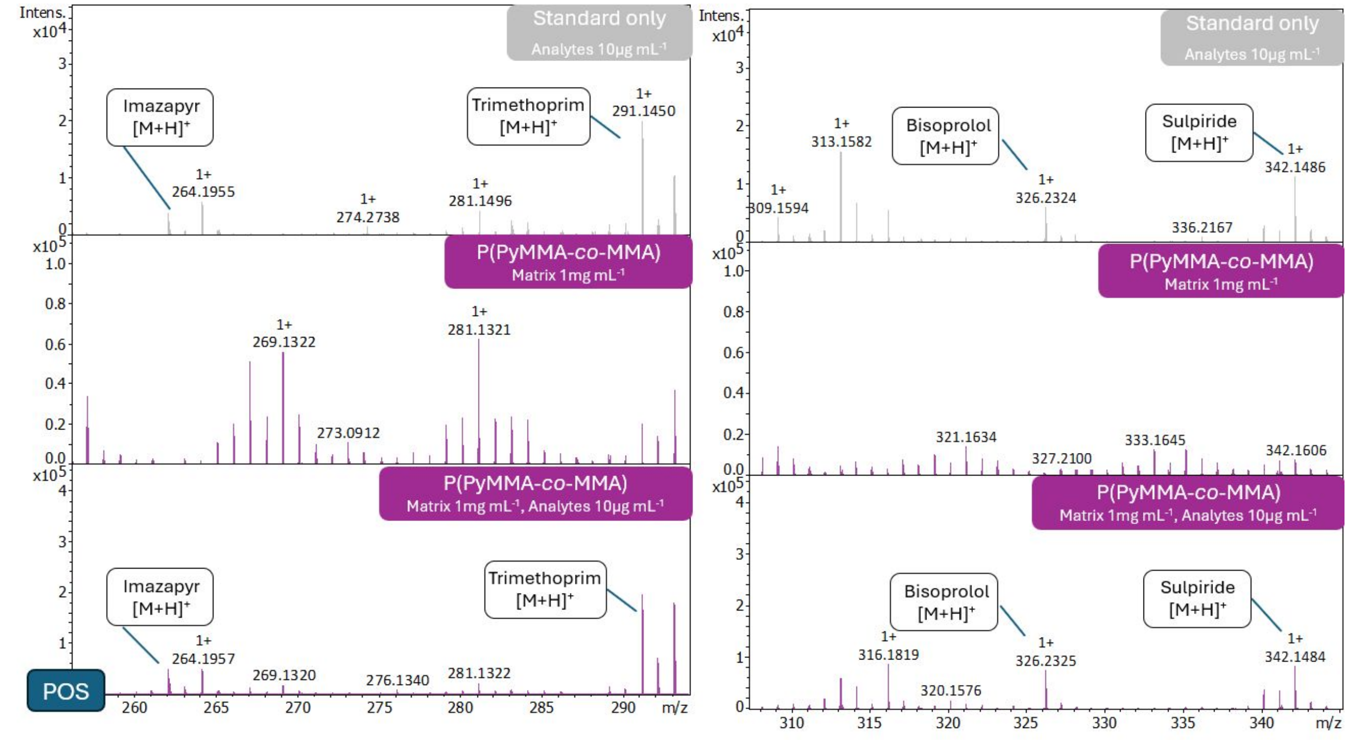

Figure S10 Positive mode MALDI-QTOF-MS spectra of Imazapyr, Trimethoprim, Bisoprolol, and Sulpiride acquired using P(PyMMA-co-MMA) (magenta, lower spectra) as matrix, along with spectra obtained without any matrix (light grey, upper spectra) and spectra containing only the matrix (magenta, middle spectra).

Table 2 contains the signal-to-noise ratio values (S/N) comparison of all tested polymers, including the commercially available matrix P3DDT. Two out of the three newly synthesized polymers namely, PPyMMA and P(PyMMA-*co*-MMA), exhibited ameliorated S/N values when compared to the commercially available P3DDT. P(PyMMA-*r*-DMAEMA) exhibited the lowest S/N values out of all polymers tested.

Table 2 Table containing the signal-to-noise (S/N) ratio for all four analytes with all four tested polymeric MALDI matrices.

Analyte Name	P3DDT S/N values	PPyMMA S/N values	P(PyMMA-*r*-DMAEMA) S/N values	P(PyMMA-*co*-MMA) S/N values
Imazapyr	12	18	10	13
Trimethoprim	33	52	16	49
Bisoprolol	7	13	6	19
Sulpiride	15	25	5	21

In Figure S11, the MS spectra of each polymer, containing only the matrix solution ("matrix only" samples) are presented under the same laser intensity (70%) and laser shots (10 bursts of 200 shots), with which comparison of analyte ionization efficiency was performed for the samples containing both the matrix and the analyte standard solution. Across all the mass spectra of the synthesized polymers, the fragment ion at m/z 215.0855 and its potential dimer at m/z 430.1716 can be observed with elevated signal intensities.

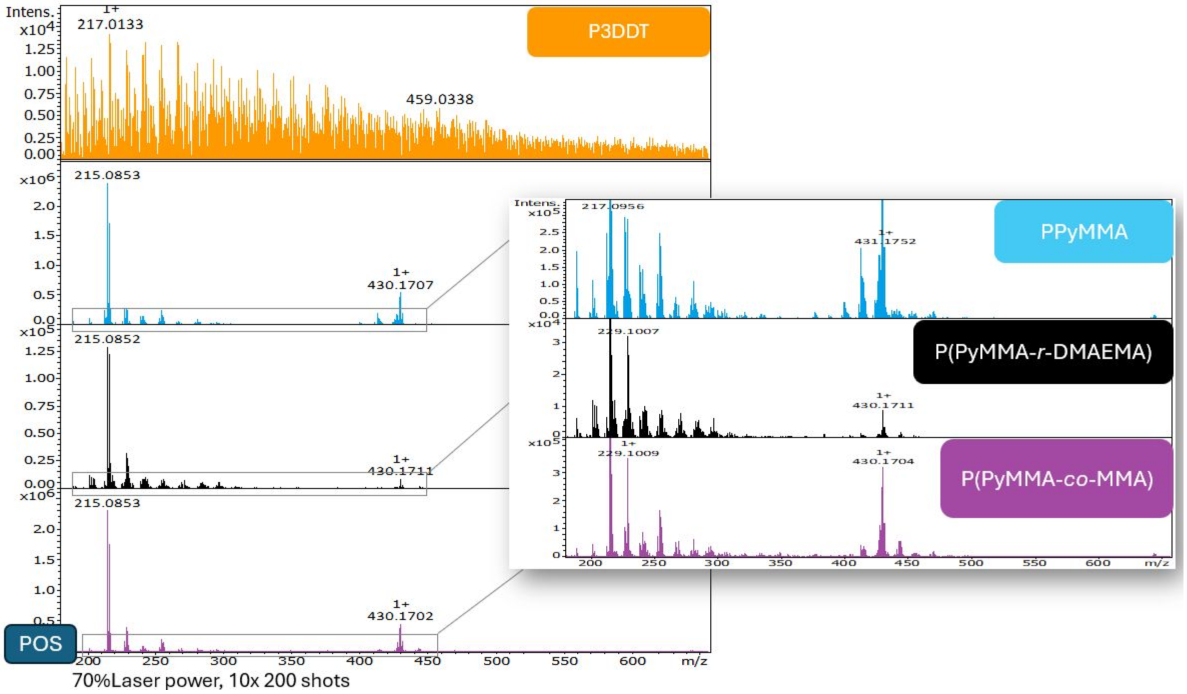

Figure S11 Positive mode MALDI-QTOF-MS spectra acquired with 70% laser power and 2.000 shots, containing only P3DDT (orange), PPyMMA (blue), P(PyMMA-r-DMAEMA) (black) and P(PyMMA-co-MMA) (magenta) as matrices, each at a concentration of 1 mg mL^{-1}.

In Figure S12, the spectra of all tested polymeric matrices from the samples containing only the matrix (“matrix only”) are presented and zoomed in the region of 214-222 m/z (left stacked spectra) and between 195-210 m/z (right stacked spectra), depicting the matrix fragment ion 215.0855 m/z, the 216.0934 m/z ion, (potentially corresponding to the $[M+H]^+$ of 215.0855 m/z, its first isotope or a contribution of both), potential pyrene fragment ion species (e.g. $[M]^+$ at m/z 202.0777), and their respective abundances.

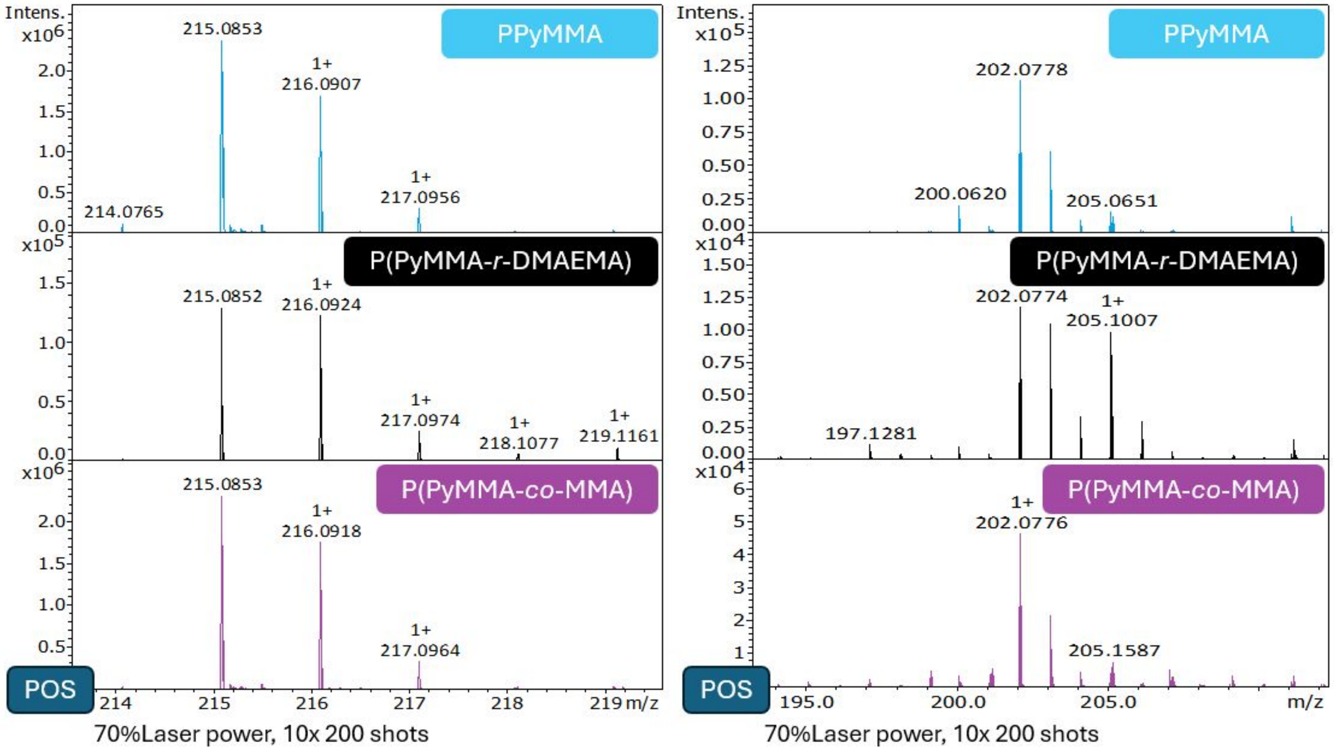

Figure S12 Positive mode MALDI-QTOF-MS spectra acquired with 70% laser power and 2.000 shots with PPyMMA (blue), P(PyMMA-r-DMAEMA) (black) and P(PyMMA-co-MMA) (magenta) as matrices, each at a concentration of 1 mg mL^{-1}. The figure depicts the fragment ion at m/z 215.0855 m/z, the ion at m/z 216.0934, potentially a $[M+H]^+$ ion of 215.0855 m/z or/and its first isotope (left spectra), as well as potential pyrene fragment species (e.g. at m/z 202.0777, pyrene $[M]^+$), (right spectra) and their respective abundances.

Thermal Properties

Chromophores with extended conjugated systems, such as pyrene, are compounds that seem to be highly stable upon thermal decomposition.[1] Furthermore, methacrylate-based polymers have been extensively studied for their thermal behavior.[2] As previously stated, we chose a methacrylic backbone in all polymers synthesized to produce polymers that could possibly preserve the amorphous morphology of the parent methacrylic backbone. Additionally, thermal stability is a crucial prerequisite for a MALDI-TOF matrix. For this reason, thermogravimetric analysis (TGA) was performed to gain insight into polymers’ decomposition patterns, followed by differential scanning calorimetry (DSC) measurements that reveal whether the amorphous morphology can be maintained. DSC thermographs for the random copolymers P(PyMMA-*co*-MMA) and P(PyMMA-*r*-DMAEMA) are not shown here, as P(PyMMA-*co*-MMA) displayed only a single glass transition temperature (Tg) around 100 °C, comparable to that of PMMA, while P(PyMMA-*r*-DMAEMA) exhibited no discernible thermal transitions up to 180 °C. The most interesting results

were obtained for homopolymers, PPyMMA. A *Tg* transition that was noticeably greater than PMMA emerged at 156 °C after the initial heating (up to 180 °C, Figure S13 green line). This finding, in combination with the extensive excimer formation documented in PL measurements, led us to the hypothesis that these macromolecules may contain crystalline regions. In a second heating scan up to 350 °C, a sharp melting transition appeared at 212 °C, followed by a subsequent exothermic transition, as shown in Figure S13.

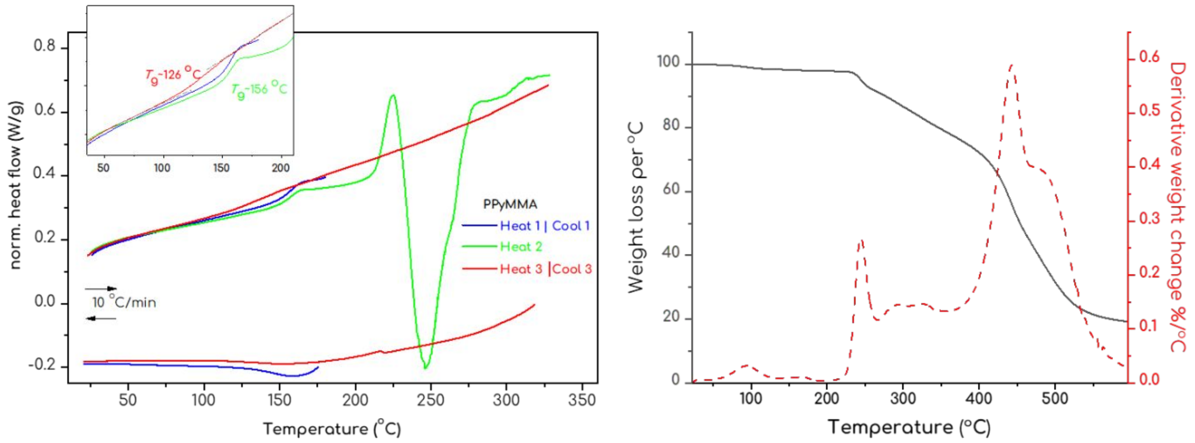

Figure S13 DSC traces, during three heating/cooling scans, of PPyMMA, along with the respective Tg values. (left) and TGA thermographs, for PPyMMA homopolymer [12k, DP=40] (right).

The acquired TGA data confirmed that the exothermic peak that follows the melting transition is generated by decomposition processes. The appearance of the curve suggests a complex decomposition mechanism, although it may be assumed that decomposition initially occurs by cleavage of the pendant side chains bearing pyrene (ether bond cleavage). Additionally, there is a 20% char residue up to 600°C, that may be attributed to high thermal stability of fused ring system in pyrene.

References

(1) Aguilar-Martínez, M.; Antonio Bautista-Martínez, J.; Rivera, E. Thermal, Optical, Electrochemical Properties and Conductivity of Pyrene Monomers. *Des. Monomers Polym.* **2008**, *11* (2), 173–186. https://doi.org/10.1163/156855508X298071.
(2) Ali, U.; Karim, K. J. Bt. A.; Buang, N. A. A Review of the Properties and Applications of Poly (Methyl Methacrylate) (PMMA). *Polym. Rev.* **2015**, *55* (4), 678–705. https://doi.org/10.1080/15583724.2015.1031377.
